# Multifunctional Hybrid Membrane-Coated Nanomotors for Magnetically Guided, Cascade-Activated Chemoimmunotherapy for Triple-Negative Breast Cancer

**DOI:** 10.1021/acsami.5c10644

**Published:** 2025-09-04

**Authors:** Man Lung Lee, Li Wang, Jack Chun Hin Chen, Ellen Ngar-Yun Poon, Dinggeng He, Hung-Wing Li

**Affiliations:** † Department of Chemistry, 26451 The Chinese University of Hong Kong, Shatin, Hong Kong SAR 999077, China; ‡ College of Life Science, 12568 Hunan Normal University, Changsha 410081, China; § School of Biomedical Sciences, The Chinese University of Hong Kong, Shatin, Hong Kong SAR 999077, China; ∥ Hong Kong Hub of Paediatric Excellence (HK HOPE), The Chinese University of Hong Kong, Shatin, Hong Kong SAR 999077, China

**Keywords:** nanomotor, immunotherapy, cell membrane coating, chemotherapy, triple-negative breast cancer

## Abstract

Here, we report a multifunctional hybrid membrane-coated nanomotor for cancer chemoimmunotherapy, which consists of mesoporous silica-coated iron oxide nanoparticles (MF) as a drug carrier, loaded with doxorubicin (DOX), l-arginine (l-arg), and glucose oxidase (GOx), and camouflaged with a hybrid of red blood cell membranes (mRBC) and cancer cell membranes (CCM). RM-GDL-MF has a cascade of catalytic reactions, where glucose is catalyzed by GOx to produce H_2_O_2_, and l-arg is oxidized by the produced H_2_O_2_ to release nitric oxide (NO), leading to self-propelled motion in order to promote the penetration of the extracellular matrix (ECM) in the tumor. The hybrid membrane provides not only stealth properties from mRBC to evade immune clearance but also tumor-orientation ability to target the tumor from the CCM. The generation of reactive oxygen species (ROS) was demonstrated to induce immunogenic cell death (ICD), thereby enhancing antitumor immune responses through the recruitment of CD8^+^ tumor-infiltrating lymphocytes (TILs), promotion of dendritic cell maturation, and reprogramming of macrophages toward the proinflammatory M1 phenotype. *In vitro* and *in vivo* experiments demonstrated that RM/R4-GDL-MF had significant tumor accumulation efficiency and antitumor efficacy. This multimodal nanomotor shows great potential for effectively treating tumors.

## Graphical abstract

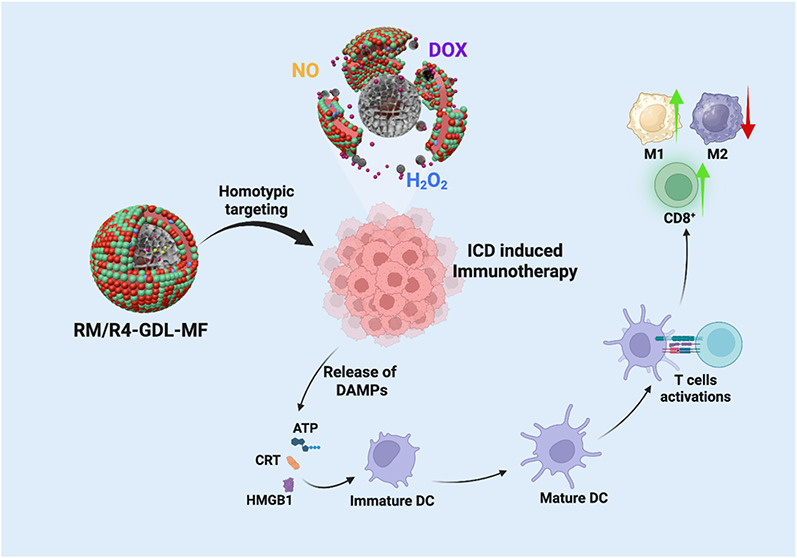

## Introduction

According to the World Health Organization, cancer is the second leading cause of death globally, accounting for nearly 10 million deaths in 2020.^1^ Despite significant advancements in cancer diagnosis and treatment, patients with solid tumors often face poor prognoses and severe side effects from conventional therapies.^1–3^ Triple-negative breast cancer (TNBC) is an especially aggressive subtype that spreads more rapidly than other forms of breast cancer.^2^ Characterized by the absence of estrogen receptor (ER), progesterone receptor (PR), and human epidermal growth factor receptor 2 (HER2), TNBC lacks specific targeted therapies, making treatment particularly challenging.^3^ The standard clinical approach for TNBC involves surgical resection followed by chemotherapy and radiotherapy.^4^ However, the effectiveness of chemotherapy is severely limited by the dense extracellular matrix (ECM) and high interstitial fluid pressure within the tumor microenvironment, which restricts drug penetration and retention, leading to systemic toxicity.^5–7^

Cell membrane-coating strategies have been extensively explored to endow nanoparticles with biomimetic functionalities. Various cell membranes, including those derived from red blood cells, platelets, white blood cells, and cancer cells, have been used to functionalize nanoparticles, imparting distinct biological advantages.^8–11^ For instance, Zhang et al. developed red blood cell (RBC)-coated nanoparticles as drug carriers, which demonstrated a prolonged blood circulation time of 39.6 h compared to 15.8 h for poly­(ethylene glycol) (PEG)-coated nanoparticles. This extended circulation is attributed to the overexpression of the CD47 protein on the RBC surface, which sends “do not eat me” signals to prevent macrophage-mediated phagocytosis. Additionally, cancer cells exhibit a strong tendency to adhere to one another, a characteristic that facilitates tumor progression.^12^ This homotypic binding property makes cancer cell membranes highly suitable for nanoparticle coating, enhancing their tumor-targeting capability.^13,14^ By fusing RBC and cancer cell membranes into a single hybrid coating, our platform subtly achieves both enhanced targeting and immune evasion.^15^

The emergence of nanomotors with autonomous motion offers a promising strategy to enhance nanoparticle penetration through the dense ECM by generating chemotactic movement.^16^ Among various types of nanomotors, chemically propelled nanomotors, which convert chemical energy into kinetic energy by utilizing endogenous chemical gradients, hold great potential for ECM penetration, particularly under the reactive oxygen species (ROS)-rich tumor microenvironment.^17^ For example, Peng et al. designed a platinum nanoparticle-based stomatocyte capable of reacting with H_2_O_2_ to generate O_2_, enabling gas-driven propulsion.^18^ However, most chemically driven nanomotors, whether metal-based or enzyme-based, produce byproducts such as H_2_, CO_2_, NH_3_, or residual metallic ions (e.g., Pt, Mg^2+^), which can be toxic to the human body.^19^ Among catalytic gas-generating reactions, the conversion of l-arginine (l-arg) to l-citrulline and nitric oxide (NO) stands out as a biocompatible process that avoids harmful side effects.^20^ Under conditions of high ROS concentrations or inducible nitric oxide synthase activity, NO is produced, generating a propulsion force that enables nanomotors to move autonomously.^21^ Additionally, NO can react with superoxide anions to form peroxynitrite (ONOO^–^), which stimulates ECM degradation by activating matrix metalloproteinases, further enhancing tumor penetration.^22^ However, a chemically propelled nanomotor suffers from incontrollable propulsion behavior on demand. In our design, l-arg is chosen to fuel the nanomotor for deep tumor penetration when combined with magnetic guidance (magnetic iron oxide core), ensuring the nanomedicine reaches the desired tumor regions.^23^

Immunogenic cell death (ICD) has recently attracted increasing attention as a mechanism to transform nonimmunogenic tumors into immunogenic ones. ICD is characterized by the release of damage-associated molecular patterns (DAMPs), such as calreticulin (CRT) exposure on the cell surface, translocation of high-mobility group protein B1 (HMGB1) from the nucleus to cytoplasm, and secretion of ATP.^24^ There are several strategies to induce ICD, among which the generation of substantial levels of ROS is one of the most common and effective. Chemodynamic therapy (CDT) is a well-established approach for ROS production, relying on Fenton or Fenton-like reactions within the tumor microenvironment to generate cytotoxic hydroxyl radicals. For example, Liu et al. developed a ROS self-supplying calcium–copper peroxide nanocomposite that continuously releases ROS through copper-mediated Fenton-like reactions, thereby eliciting robust DAMP release and promoting ICD.^25^ These signals collectively stimulate dendritic cell (DC) maturation, facilitate T cell activation, and promote the repolarization of tumor-associated macrophages from the immunosuppressive M2 phenotype to the proinflammatory M1 phenotype. Liu et al. constructed a ROS self-generating zinc–copper bimetallic peroxides, which generates a large amount of ROS and induces ICD, and showed significant tumor-inhibiting efficacy when synergized with PD-L1 immune checkpoint inhibitor. Thus, inducing ICD represents a promising strategy for activating the host immune response against cancer.^26,27^

In this study, we developed a multifunctional hybrid membrane-coated mesoporous silica-iron oxide nanoplatform (RM/R4-GDL-MF) loaded with l-arg, glucose oxidase (GOx), and doxorubicin (DOX) for synergistic cancer chemoimmunotherapy (Scheme 1). This multifunctional platform (RM/R4-GDL-MF) integrates the l-arg–driven nanomotor, which is magnetically guided for enhanced tumor penetration, with a hybrid membrane that combines the targeting benefits of cancer cell membranes and the immune evasion properties of red blood cell (RBC) membranes. To enhance biocompatibility and tumor-targeting capability, we fused RBC membranes with cancer cell membranes to create a hybrid membrane-coated nanoparticle. The RBC membrane helps the drug carrier evade immune clearance, while the cancer cell membrane facilitates homotypic tumor targeting. The large surface area of mesoporous silica affords high-efficiency loading of l-arg, GOx, and DOX. The hybrid membrane coating helps prevent DOX from leaking during blood circulation, accomplishing controlled release of DOX in tumor sites, which significantly reduces the systemic toxicity of the chemotherapeutic drug. Under the tumor microenvironment, GOx catalyzes the oxidation of intracellular glucose, producing cytotoxic hydrogen peroxide (H_2_O_2_), which induces tumor inflammation and starvation. The resultant H_2_O_2_, together with NO generated from l-arg, orchestrates a synergistic immunotherapeutic response by repolarizing tumor-associated macrophages from the M2 to the M1 phenotype, promoting dendritic cell maturation, and recruiting CD8^+^ tumor-infiltrating lymphocytes, thereby enhancing antitumor immunity. Additionally, l-arg serves a dual function: it propels the nanomotor through the ECM while generating NO, which subsequently reacts with ROS to form ONOO^–^, exhibiting strong tumor-killing activity. The iron oxide core that exhibits magnetic properties enhances the tumor accumulation of the nanomedicine as guided by an external magnetic field. We here demonstrated that the synergistic integration of the hybrid membrane, the multifunctional nanomotor mechanism, and the chemoimmunotherapeutic agents provides a potent platform for enhanced treatment of triple-negative breast cancer.

**Scheme 1:**
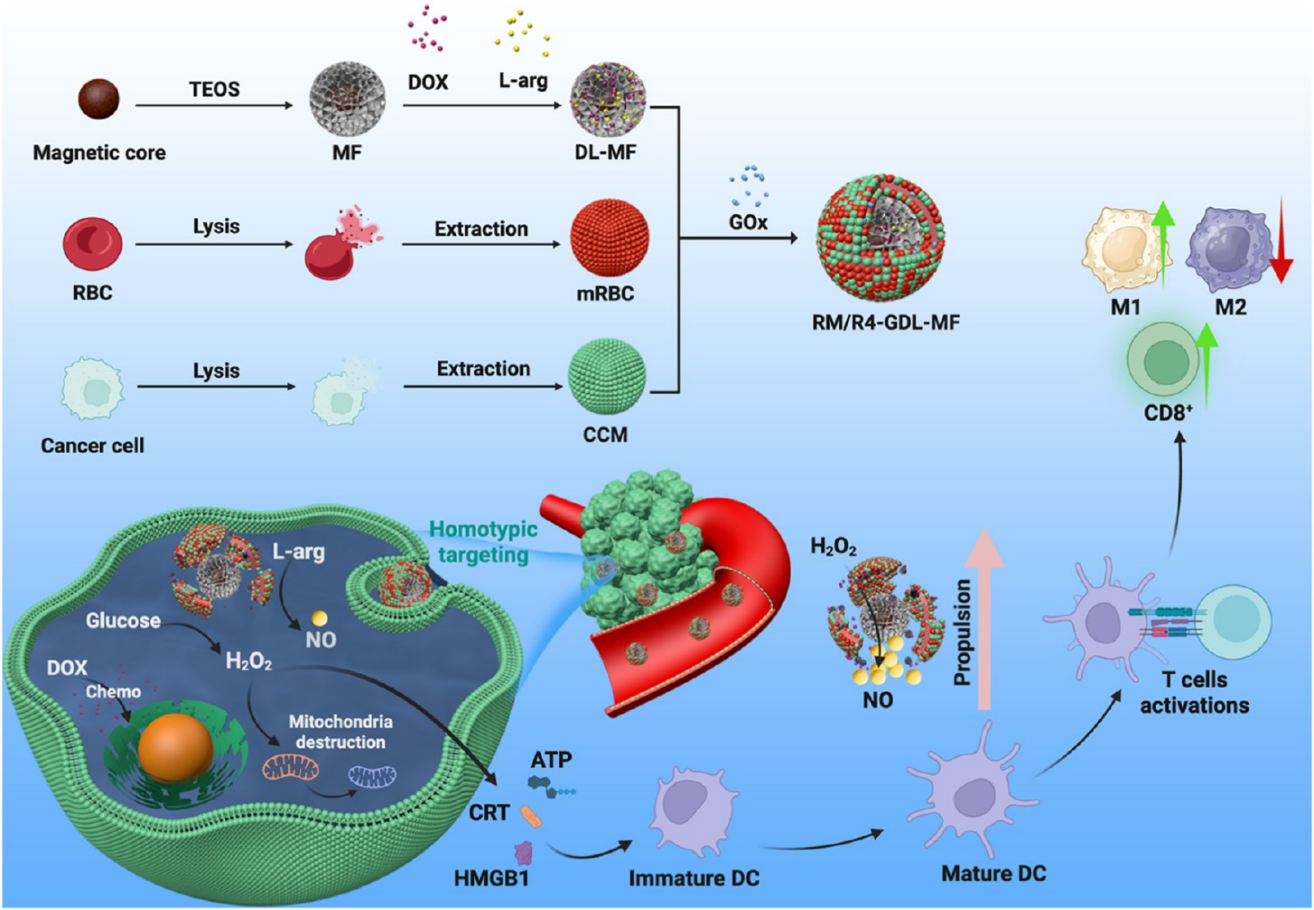
Schematic Illustration of the Synthesis of RM/R4-GDL-MF and Its Antitumor Mechanism

## Results and Discussion

### Preparation and Characterization of RM-GDL-MF

The synthesis of RM-GDL-MF is illustrated in Scheme 1. First, Mesoporous silica-iron oxide (MF) was synthesized through the Stöber process, where a silica shell was coated onto an iron oxide core under alkaline conditions. Transmission electron microscopy (TEM) revealed a uniform iron oxide core and mesoporous silica shell structure (Figure 1A). Dynamic light scattering showed an average size of 128 nm for the MF nanoparticles (Figure S1 **)**. The MF was further modified to NH_2_-MF and COOH-MF, as confirmed by ζ-potential and Fourier transform infrared spectroscopy analyses (Figures S2 and S3). In the FTIR spectrum, NH_2_-MF exhibited a characteristic N–H bending peak at 1643 cm^–1^, while the COOH group displayed two distinct peaks at 1419 cm^–1^ (O–H bending) and 1296 cm^–1^ (C–O stretching). The spectrum of COOH-MF contained both the N–H peak (1643 cm^–1^) and the COOH peaks (1419 cm^–1^ and 1296 cm^–1^), confirming the successful modification of MF to COOH-MF. The ζ-potential showed that the negatively charged MF (−39.8 mV) changed to positively charged NH_2_-MF (+29.9 mV) through APTES treatment and then further modified to COOH-MF (−52.7 mV) by reacting with succinic anhydride. The MF was then loaded with l-arg and DOX to create DL-MF, and GOx was also loaded into DL-MF after removing any unloaded DOX and l-arg. The morphologies of DL-MF and GDL-MF remained unchanged compared to MF, as shown by TEM (Figure S4). In this study, MDA-MB-231/4T1 cell membrane and red blood cell membrane were chosen to target breast cancer. The sonication method was used to prepare RM-GDL-MF. A thin layer of the membrane could be observed on the nanoparticles under TEM (Figure 1B). The mean sizes of MF, DL-MF, and RM-GDL-MF were determined to be 128, 130, and 136 nm, respectively, illustrating that the successful coating of membranes altered the nanoparticle morphology. Their ζ-potentials were −52.7, −16.5, and −32.8 mV (Figure 1C), respectively, indicating the successful loading of drugs, as confirmed by FTIR analysis (Figure S5). The characteristic peaks of DOX at 2978 cm^–1^ and l-arg at 868 cm^–1^ and 1358 cm^–1^ were observed in the FTIR spectrum of DL-MF, confirming the successful loading of l-arg and DOX. Similarly, GOx exhibited two characteristic peaks at 1654 cm^–1^ and 1560 cm^–1^, which were also present in the spectrum of GDL-MF, indicating the successful incorporation of GOx. The loading efficiency (LE%) and encapsulation efficiency (EE%) of DOX and l-arg on the MF were calculated based on the unloaded drugs, where the LE% and EE% of DOX were 15.8% and 95.2%, respectively, while the LE% and EE% of l-arg were 74.8% and 54.8% (Figure 1D). Furthermore, SDS-PAGE analysis of a series of membrane proteins and GOX markers confirmed the successful coating of GOx and membrane proteins onto the DL-MF (Figure 1E). In Figure S8, confocal laser scanning microscopy of RM-GDL-MF, with DiI-labeled MF and DiD-labeled hybrid membrane, revealed the overlapping signals, indicating the successful coating of membranes onto MF. Furthermore, thermogravimetric analysis (TGA) was performed to verify the successful stepwise loading of therapeutic agents and the coating of the hybrid membrane. The TGA analyses showed progressive increases in the organic content corresponding to each modification (Figure S9). Compared with MF, DL-MF exhibited an additional ∼5 wt % mass loss, attributed to the decomposition of DOX and l-arg. GDL-MF showed a further ∼12 wt % loss, consistent with the presence of GOx. R4-GDL-MF displayed an additional ∼15 wt % loss, arising from the lipid and protein components of the hybrid membrane coating. The residual mass at 800 °C was predominantly the inorganic MF core. After 24 h of incubation in acidic environments (pH 5.5, 500 μM glucose), the hybrid membrane structure was disrupted by the flux of NO production under the cascade reaction of H_2_O_2_ and l-arg (Figure 1F), allowing for the efficient release of the loaded DOX in the acidic tumor microenvironment. The drug release was tested under 4 different conditions, and the release of DOX in pH 5.5 and 500 μM glucose PBS buffer was the most efficient (Figure 1G). Importantly, the coating of hybrid membranes helped prevent drug leakage, as DOX release from DL-MF was higher than that from RM-GDL-MF regardless of pH, as shown in Figure S10. The generation of H_2_O_2_ by RM-GDL-MF was quantified over 24 h after incubation in different media at 37 °C, revealing large amounts of H_2_O_2_ produced in the presence of glucose, with a more prominent production under acidic conditions (Figure 1H). The produced H_2_O_2_ further reacts with loaded l-arg to generate NO gas, which serves as the propulsion force of the nanomotor. The release of NO over a 24 h period was quantified using an NO assay kit, and more NO was produced in the presence of glucose (Figure 1I). Moreover, hemolysis experiments were conducted to evaluate the biosafety of RM/R4-GDL-MF. The positive control (DI water) exhibited a red supernatant and strong absorbance at 545 nm, indicating the significant hemolysis of red blood cells. In contrast, no noticeable hemolysis was observed in the treatment groups or the negative control, demonstrating the excellent biocompatibility of RM/R4-GDL-MF (Figure S11). These results suggest that RM-GDL-MF was successfully synthesized, with high loading efficiency of DOX and l-arg, and successful coating of a hybrid membrane composed of RBC and CCM. The drug release quantification also confirms that the release of DOX and the generation of H_2_O_2_ are more efficient in the presence of glucose and an acidic environment. The generation of NO in the presence of glucose also indicates the successful cascade reaction between GOx and l-arg, in which produced H_2_O_2_ catalyzes the conversion to NO gas.

**Figure 1:**
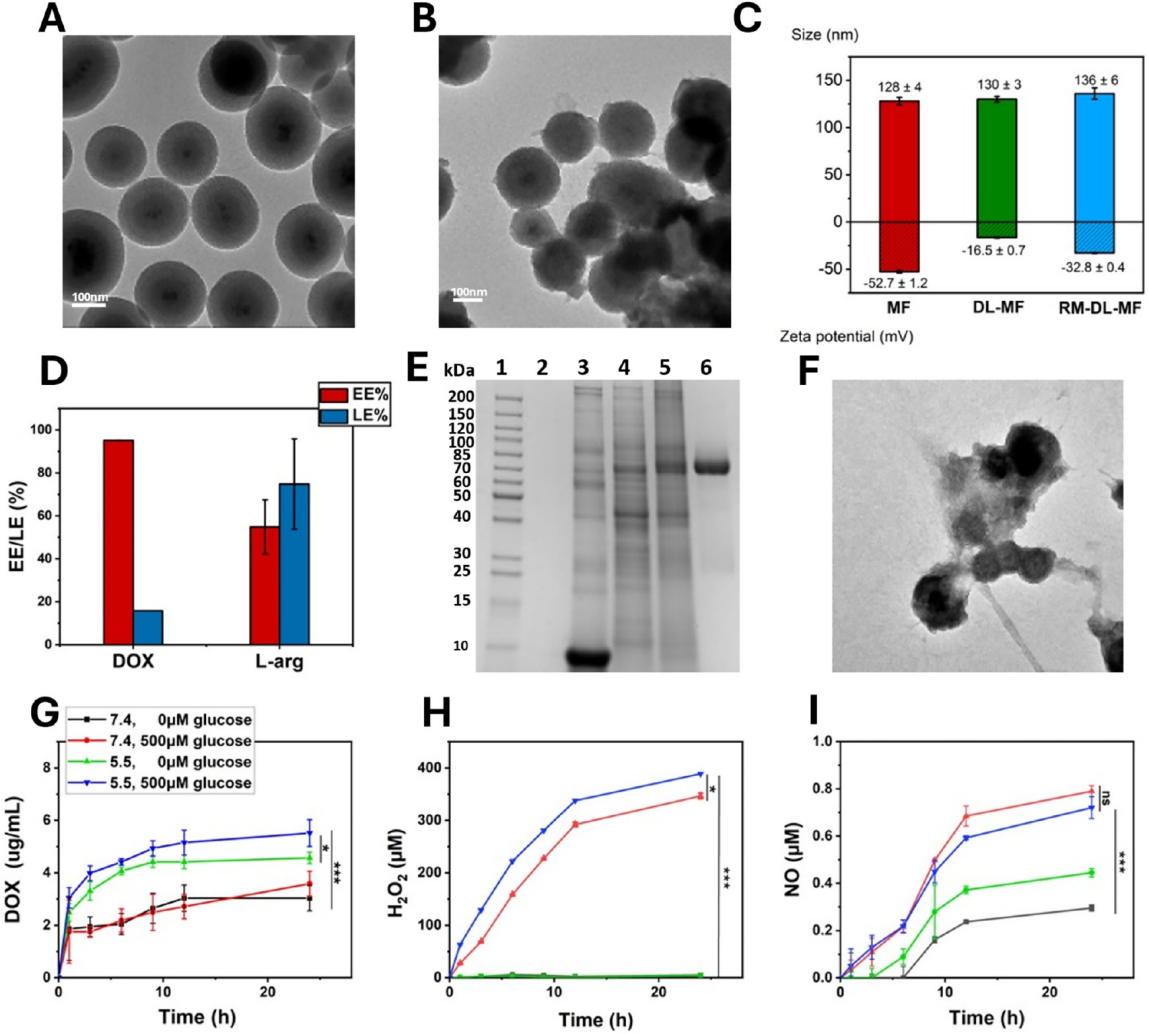
Characterization of RM-GDL-MF. (A) and (B) TEM images of MF and RM-GDL-MF. (C) Size and ζ-potential of MF, DL-MF, and RM-GDL-MF. (D) Encapsulation and loading efficiency of DOX and l-arg. (E) SDS-PAGE of RM-DL-MF (1) protein ladder, (2) MF, (3) RBC, (4) MDA, (5) RM-DL-MF, and (6) GOX. (F) RM-GDL-MF incubated in 500 μM glucose PBS (pH 5.5). (G) DOX release, (H) H_2_O_2_ release, and (I) NO release of RM-GDL-MF in different pH and glucose content for 24 h at 37 °C.

### Motion Behavior of Nanomotor

The motion behavior of the nanomotor was examined in a glucose solution. The autonomous movement is driven by the conversion of l-arg to l-citrulline and NO gas, which occurs under high ROS and iNOS concentrations commonly found in the highly inflammatory tumor microenvironment. As illustrated in Figure 2A, the schematic diagram shows that RM-GDL-MF reacts with glucose in the medium to generate H_2_O_2_, which then reacts with the loaded l-arg to produce NO gas for nanomotor propulsion. The nanomotor’s trajectory was recorded by nanoparticle tracking analysis (NTA), and the mean square displacement (MSD) was measured. The motion tracks of the nanomotor in the presence and absence of glucose were plotted by using ImageJ software. The trajectories reveal that RM-GDL-MF exhibited Brownian motion in the absence of glucose, but autonomous motion in the 500 μM glucose solution, supporting self-propulsion of the nanomotor (Figure 2B). The motion of RM-GDL-MF was observed in the Supporting videos, showing the particles moving in 0 and 500 μM glucose solutions (S1 and S2 videos). According to the NTA analysis, the diffusion coefficient of RM-GDL-MF increased from 0.84 μm^2^/s in 0 μM glucose to 2.3 μm^2^/s in 500 μM glucose, indicating a significant velocity increase (Figure 2C). Furthermore, the MSD of RM-GDL-MF was analyzed according to the trajectory shown in Figure 2B. The two-dimensional MSD showed a linear plot with time intervals, and the MSD versus Δ*T* indicated that the motion behavior was enhanced in the presence of glucose (Figure 2D). These results demonstrated that the cascade reaction between GOx and l-arg successfully generates a propulsive force by producing H_2_O_2_ and NO bubbles in the presence of glucose.

**Figure 2:**
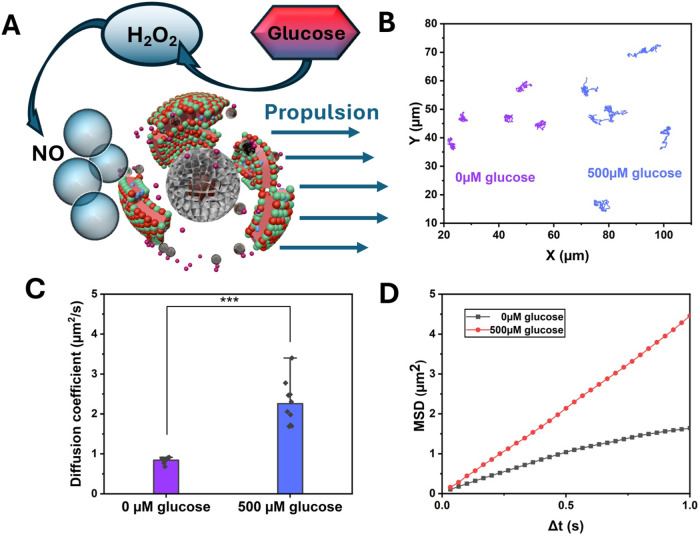
Characterization of the motion behavior of the nanomotor. (A) Schematic illustration of the autonomous motion of the nanomotor. (B) Trajectory and (C) Diffusion coefficients of RM-GDL-MF in 0 and 500 μM glucose solution. (D) MSDs of RM-GDL-MF in 0 and 500 μM glucose solution.

### Antitumor Effect of RM-GDL-MF *In Vitro*

First, the cellular uptake of RM-GDL-MF was studied in MDA-MB-231, RAW264.7, and 4T1 cells using confocal laser scanning microscopy. RAW264.7 cells were used to verify that the RBC membrane could evade immune attack, while 4T1 cells were used to verify the specific targeting of the MDA-MB-231 membrane of RM-GDL-MF to MDA-MB-231 cells. Among all groups in MDA-MB-231 cells, RM-GDL-MF exhibited the strongest fluorescence intensity, attributable to its nanomotor properties and the targeting ability of the cancer cell membrane coating (Figure 3A,B). GDL-MF showed 70% stronger uptake than DL-MF due to GOx-generated H_2_O_2_, which enhanced nanomotor propulsion and promoted active nanoparticle internalization. The RAW264.7 cells showed fluorescence intensity was 3 times weaker than that in MDA-MB-231 cells, revealing no significant uptake of RM-GDL-MF in macrophages. Furthermore, cellular uptake studies in 4T1 cells revealed a 4-fold lower fluorescence intensity compared to MDA-MB-231 cells, highlighting the specific targeting ability of RM-GDL-MF toward MDA-MB-231 cells. Then, the *in vitro* cytotoxicity of RM-GDL-MF was further evaluated in both MDA-MB-231 cells and L929 cells. The cytotoxic effects of RM-GDL-MF were assessed by using live/dead staining and an MTT assay. After 24 h of incubation, almost no live cells were observed in the GDL-MF-treated and RM-GDL-MF-treated groups, indicating obvious toxicity toward MDA-MB-231 cells (Figure 3C). The results from the MTT assay were also consistent (Figure 3D), showing concentration-dependent toxicity in the GDL-MF-treated and RM-GDL-MF-treated groups. Cells treated with RM-GDL-MF were less viable than the GDL-MF-treated groups, with the survival rate dropping from 36% to 8%, while exhibiting minimal toxicity toward normal cells, which maintained high survival rates even at increased concentrations (Figure S12). The results indicate that RM-GDL-MF could specifically kill cancer cells without causing significant damage to healthy cells. Additionally, DL-MF also exhibited considerable cytotoxic effects compared with the MF group, with survival rates of 73% and 91%, respectively. Next, we studied the apoptosis rate by flow cytometry. Consistent with the viability tests, GDL-MF and RM-GDL-MF both induced strong cell apoptosis, with 39% and 53%, respectively, while MF and DL-MF showed less significant cell apoptosis, with 4% and 21%, respectively (Figure 3E). These analyses indicate that the RM-GDL-MF-mediated cascade reaction exhibits effective cancer-killing effects compared with the control groups. At the same time, the hybrid membrane prevents the nanoparticles from entering normal healthy cells, which is supported by the high survival rate of the RM-GDL-MF-treated normal cells.

**Figure 3:**
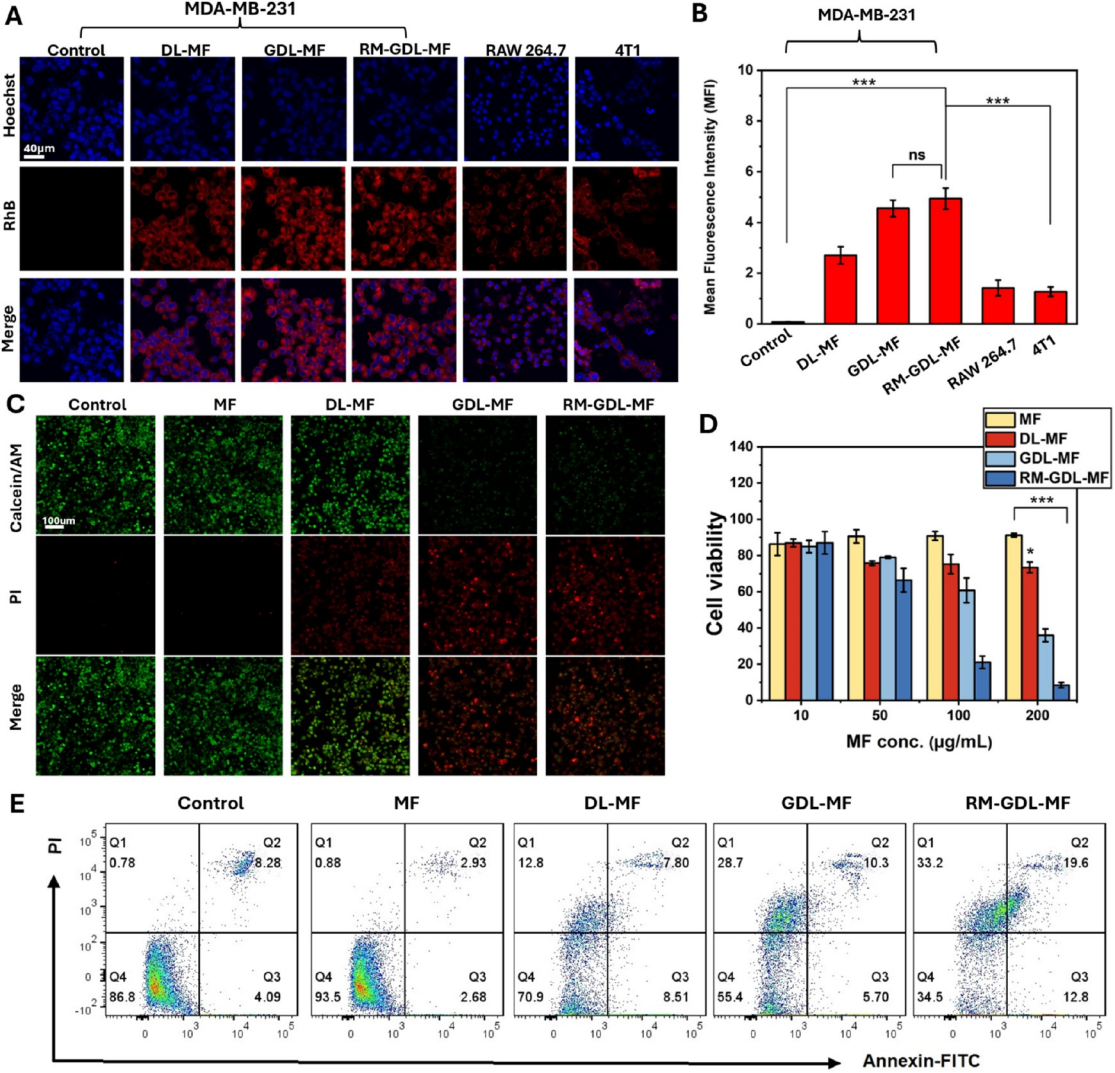
*In vitro* cellular uptake and antitumor efficacy. (A) Cellular uptake of different nanoparticles (NPs) (DL-MF, GDL-MF, RM-GDL-MF) in MDA-MB-231 cells and Cellular uptake of RM-GDL-MF in RAW264.7 cells, and 4T1 cells. Scale bar: 40 μm. (B) Corresponding fluorescence intensity. (C) Live/dead staining of MDA-MB-231 cells after different NPs treatments (MF, DL-MF, GDL-MF, and RM-GDL-MF). Scale bar: 100 μm. (D) MTT assay of MDA-MB-231 cells after different NP treatments. (E) Measurement of cell apoptosis by annexin V/PI double staining.

### *In Vitro* Intracellular ROS and NO Generation

Encouraged by the results of the H_2_O_2_ and NO release assays in Figure 1H,I, we next investigated the intracellular generation of H_2_O_2_ and NO in MDA-MB-231 cells. The GOx encapsulated in MF generates ROS by converting glucose into H_2_O_2_, which can be detected using dichlorodihydrofluorescein diacetate (DCFH-DA). This fluorescent dye is oxidized by intracellular ROS to emit green fluorescence (Figure 4A). Both the GDL-MF-treated and RM-GDL-MF-treated groups exhibited strong green fluorescence compared with the control groups, with the RM-GDL-MF-mediated cascade reaction slightly increasing intracellular ROS levels relative to GDL-MF. We then examined the cascade catalytic generation of NO, mediated by H_2_O_2_. The l-arg encapsulated in the nanoparticles reacts with the excess ROS, and the H_2_O_2_ produced by GOx further accelerates the conversion of l-arg to NO. This process was detected using Diaminofluorescein-FM diacetate (DAF-FM-DA), a cell-permeable fluorescent probe that emits green fluorescence upon interacting with intracellular NO (Figure 4B). Cells treated with DL-MF, GDL-MF, and RM-GDL-MF displayed significantly stronger green fluorescence compared to the control group and MF. Among these, DL-MF produced the weakest fluorescence intensity as both GDL-MF and RM-GDL-MF generated higher levels of H_2_O_2_, thereby accelerating the conversion of l-arg to NO. Notably, RM-GDL-MF-treated cells exhibited a stronger green fluorescence signal than those treated with GDL-MF. These results demonstrate that the RM-GDL-MF-mediated cascade reaction efficiently generates H_2_O_2_ and NO *in vitro*.

**Figure 4:**
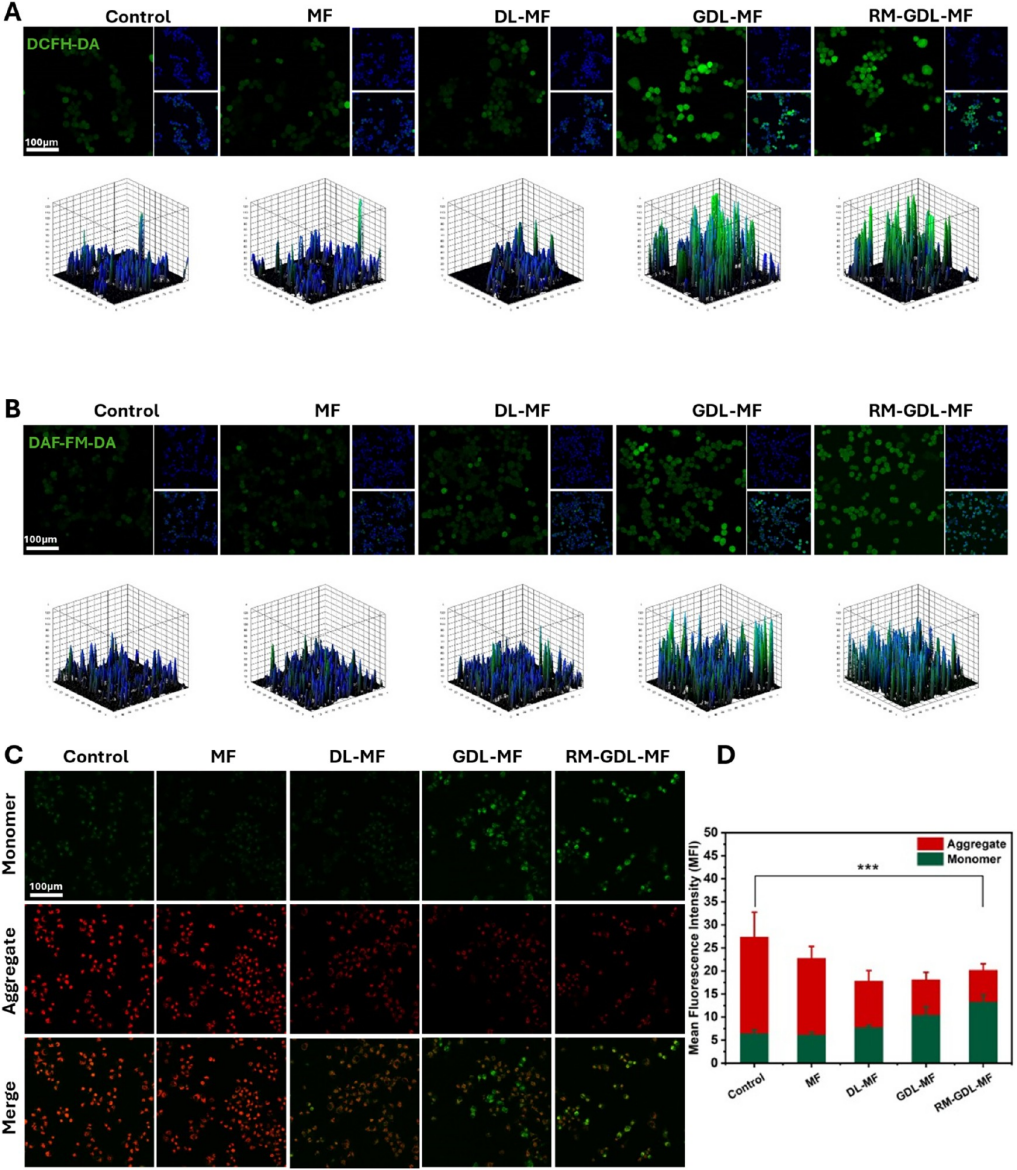
*In vitro* ROS/NO generation and mitochondria disruption. (A) DCFH-DA-stained MDA-MB-231 cells after exposure to different NPs (MF, DL-MF, GDL-MF, and RM-GDL-MF) for 4 h. (B) DAF-FM-DA-stained MDA-MB-231 cells after exposure to different NPs for 4 h. (C) JC-1-stained MDA-MB-231 cells after exposure to different NPs for 4 h. (D) Corresponding mean fluorescence intensity of JC-1-stained MDA-MB-231 cells after different NP treatments. Scale bar: 100 μm.

As the primary energy source of cells, mitochondria are particularly vulnerable to elevated ROS levels.^28^ ROS-induced mitochondrial damage was examined by using JC-1 staining. MDA-MB-231 cells treated with GDL-MF and RM-GDL-MF exhibited strong green fluorescence, reflecting mitochondrial damage and low membrane potential, whereas other nanoparticle-treated groups showed mainly red fluorescence, indicative of intact, healthy mitochondria (Figure 4C). Notably, RM-GDL-MF treatment resulted in approximately 30% higher green fluorescence intensity compared to GDL-MF, indicating a greater conversion from red aggregates to green monomers and thus more severe mitochondrial membrane depolarization (Figure 4D). These findings indicate that both GDL-MF and RM-GDL-MF significantly reduce mitochondrial membrane potential compared with the control groups, demonstrating compromised mitochondrial integrity.

### *In Vitro* Immunogenic Cell Death of Tumor Cells

We assessed the potential of RM-GDL-MF to induce ICD, beginning with an evaluation of intracellular HMGB1 localization. In the control group, the HMGB1 fluorescence was predominantly nuclear. However, upon treatment with GDL-MF and RM-GDL-MF, a significant reduction of nuclear HMGB1 fluorescence was observed (Figure 5A). Notably, the fluorescence intensities of both RM-GDL-MF and GDL-MF were comparable and approximately three times lower than that of the control group (Figure 5B). Importantly, increased HMGB1 fluorescence appeared in the cytoplasm, confirming translocation of HMGB1 from the nucleus. Additionally, ATP secretion, another hallmark of ICD, correlated well with HMGB1 release (Figure S13). To further evaluate the ICD, MDA-MB-231 cells incubated with nanoparticles were stained with the CRT antibody. Unlike HMGB1, CRT fluorescence was nearly undetectable in untreated cells but markedly increased after treatment with GDL-MF and RM-GDL-MF, confirming CRT translocation to the cell surface (Figure 5C). Notably, the fluorescence intensity of RM-GDL-MF was twice that of GDL-MF (unlike the trend observed with HMGB1) and approximately four times higher than the control group (Figure 5D). Collectively, these results indicate that RM-GDL-MF promotes CRT exposure and facilitates HMGB1 and ATP release, effectively inducing ICD in tumor cells.

**Figure 5:**
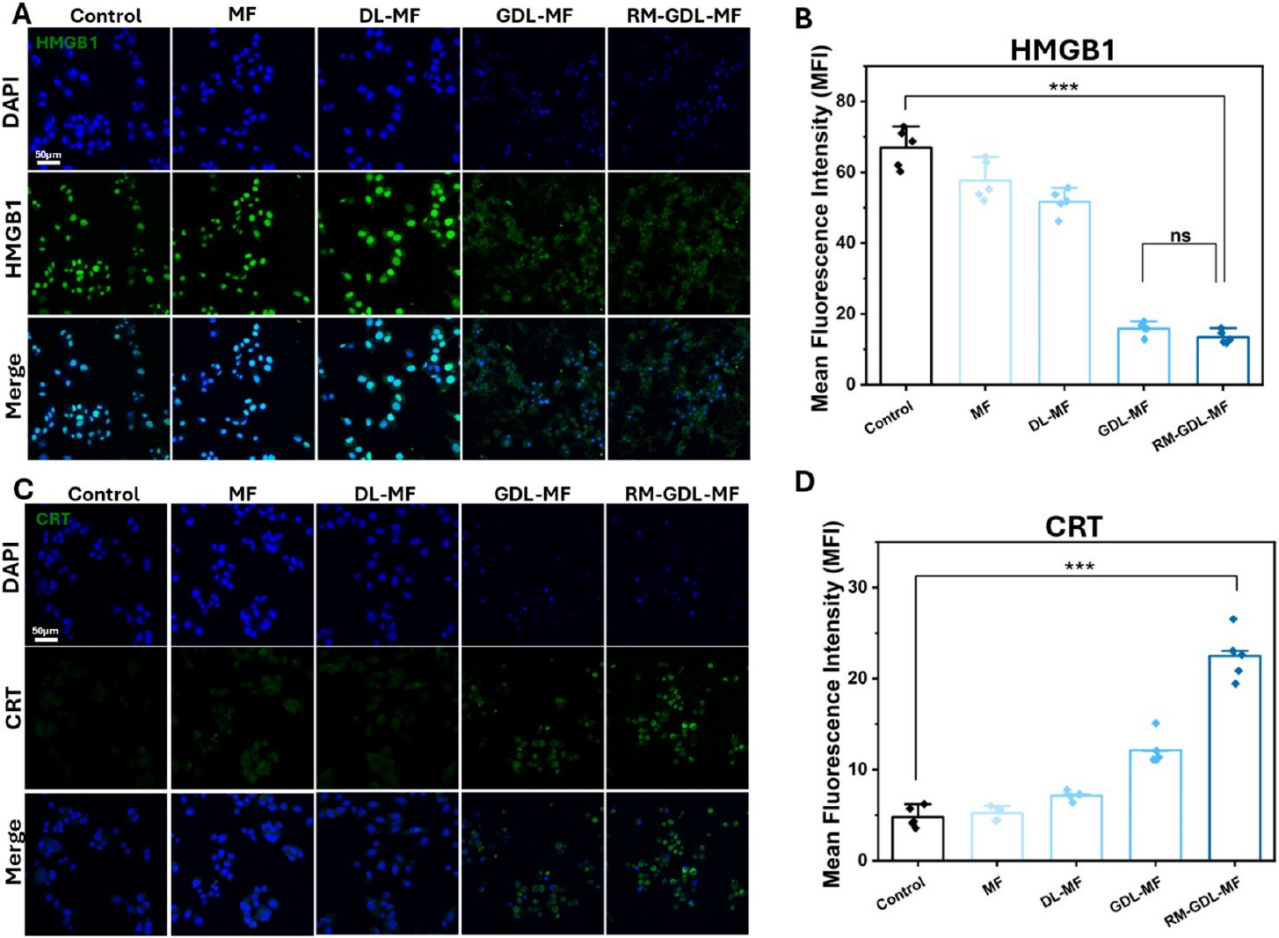
*In vitro* NPs induced ICD. (A) Fluorescence staining of HMGB1 and (B) corresponding mean fluorescence intensity of HMGB1 in MDA-MB-231 cells after different NPs treatments. (C) Fluorescence staining of CRT and (D) corresponding mean fluorescence intensity of CRT in MDA-MB-231 cells after different NPs treatments. Scale bar: 50 μm.

### *In Vivo* Biodistribution

Encouraged by the excellent antitumor efficacy of RM-GDL-MF *in vitro*, we further evaluated its therapeutic potential *in vivo*. First, we established a 4T1 xenograft mouse model to investigate the biodistribution of the nanoparticles. To enhance tumor-targeting capability, we used a 4T1 cell membrane, generating R4-GDL-MF. TEM analysis confirmed that R4-GDL-MF maintained a similar morphology to RM-GDL-MF and exhibited comparable cytotoxicity against 4T1 cancer cells, as demonstrated by the MTT assay (Figures S14 and S15). Next, we labeled R4-GDL-MF with DiR dye and administered the nanoparticles via intravenous injection. As previously mentioned, the iron oxide core of MF functions as a secondary nanomotor, facilitating nanoparticle accumulation at the tumor site under the influence of an external magnetic field. The magnetic properties of MF were evaluated by using a magnet. When exposed to a magnetic field, MF nanoparticles exhibited strong magnetic responsiveness, aggregating on the side closest to the magnet (Figure S16). 4T1 tumor-bearing mice (tumor volume ∼100 mm^3^) were randomly divided into two groups: R4-GDL-MF and R4-GDL-MF with magnetic field (M). To assess biodistribution, fluorescence intensities were measured at 0, 4, 8, 12, and 24 h postinjection using an *In Vivo* Imaging System (IVIS). After 24 h, the mice were sacrificed and their organs and tumors were collected for further analysis. As shown in Figure 6A,B, the R4-GDL-MF + M treatment group exhibited significantly stronger fluorescence intensity than only the R4-GDL-MF group, confirming that the applied magnetic field enhanced nanoparticle accumulation at the tumor site. *Ex vivo* imaging of collected organs and tumors at 24 h further supported these findings (Figure 6C). Consistently, fluorescence intensity in the tumors of the R4-GDL-MF + M treatment group was about twice as high as in the R4-GDL-MF only group (Figure 6D), while no significant differences were observed in fluorescence intensity across major organs between the two groups. These results demonstrate that the iron oxide core of the nanoparticles can be actively guided by a magnetic field, thereby enhancing nanoparticle accumulation at the tumor site.

**Figure 6:**
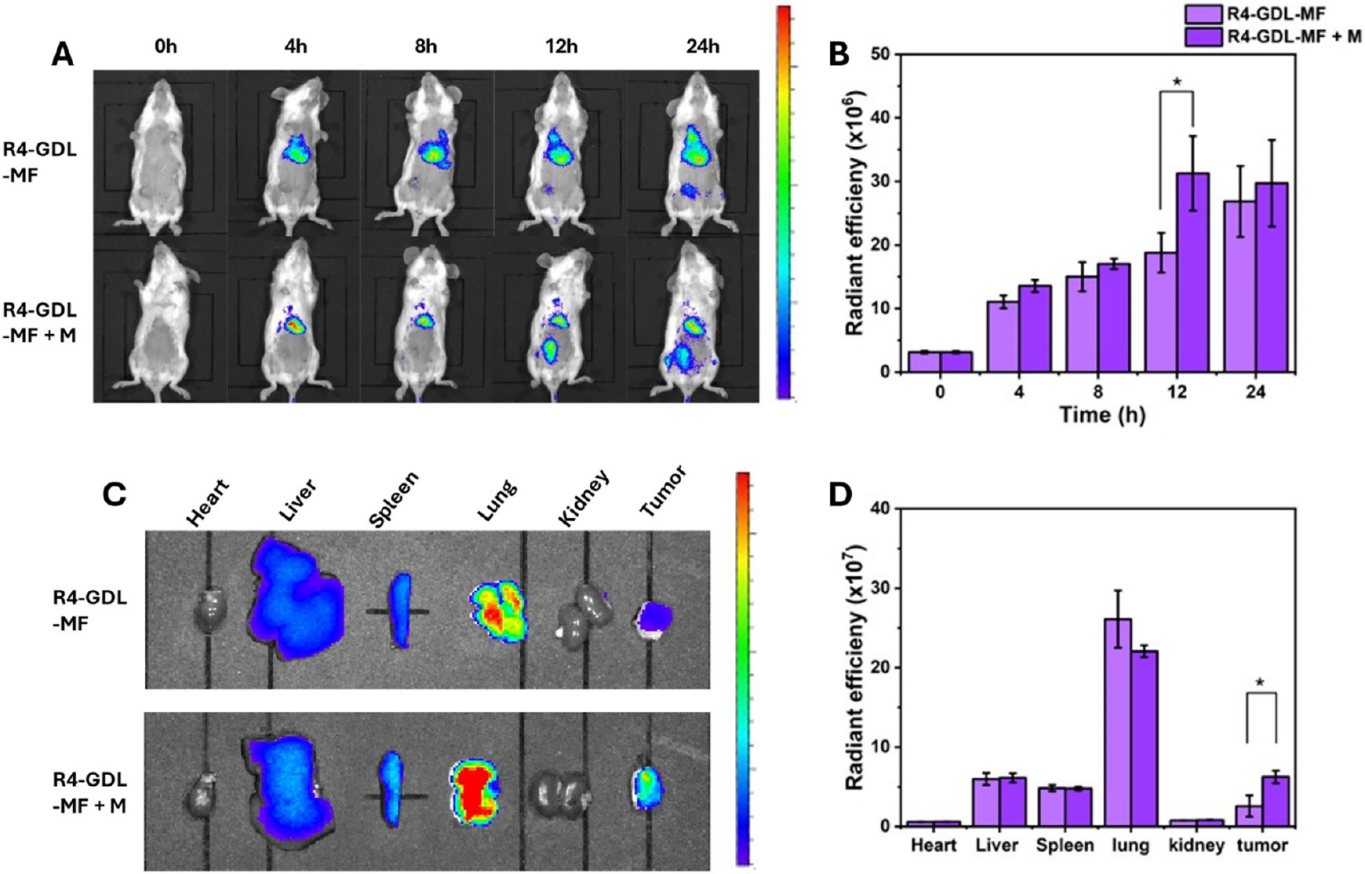
*In vivo* biodistribution. (A) Fluorescence imaging from IVIS and (B) the corresponding quantitative analysis of tumor over time with different treatments (R4-GDL-MF, R4-GDL-MF + M) (*n* = 3). (C) *ex vivo* fluorescence imaging of organs (heart, liver, spleen, and kidneys) and tumor after 24 h. (D) Corresponding quantitative analysis of the fluorescence intensity of different organs and tumors.

### *In Vivo* Antitumor Efficacy

We next evaluated the tumor inhibition efficacy of different nanoparticles (NPs) *in vivo*. 4T1 tumor-bearing mice with tumor volumes of approximately 100 mm^3^ were randomly divided into six treatment groups: PBS, DOX, DL-MF, GDL-MF, R4-GDL-MF, and R4-GDL-MF + M. Seven days after tumor inoculation, mice received intravenous (iv) injections of their respective treatments on days 0, 2, 4, and 6 (Figure 7A). Tumor volume and body weight were monitored daily, and tumors were collected after 14 days for weight measurement and immune evaluation. As shown in Figure 7B, the R4-GDL-MF + M group exhibited the most significant tumor growth inhibition with the lowest tumor weight among all groups (Figure 7C). Upon the addition of the magnetic field, more prominent antitumor effects were observed, which proved that more nanoparticles were accumulated in the tumor with magnetic guidance. The reduction in tumor volume became increasingly pronounced with repeated injections (Figure 7D,E). It is worth mentioning that the tumor volume and weight of the DL-MF-treated group were comparable to those of the GDL-MF group, likely due to the potent antitumor activity of DOX. In addition to its well-established chemotherapeutic effect, DOX has been widely reported to induce ROS generation and trigger ICD, thereby enhancing antitumor immunity. This dual mechanism may contribute to the strong therapeutic efficacy observed in the DL-MF group.^29,30^ Additionally, no significant changes in body weight were observed across treatments (Figure S17), indicating good biocompatibility of R4-GDL-MF for cancer therapy. The robust tumor inhibition achieved by R4-GDL-MF is attributed to its superior tumor-targeting capability and multimodal therapeutic action. To further validate its antitumor efficacy, hematoxylin and eosin (H&E) staining and terminal deoxynucleotidyl transferase dUTP nick-end labeling (TUNEL) assays were performed to assess tumor tissue damage and apoptosis (Figure 7F). Histological analysis verified that treatment with the R4-GDL-MF + M treatment group possessed the most extensive tumor damage and apoptosis, consistent with its strong therapeutic effect. Overall, these findings underscore the efficacy of our multifunctional nanoparticle system in achieving precise tumor targeting, enhanced penetration, and potent antitumor immunity.

**Figure 7:**
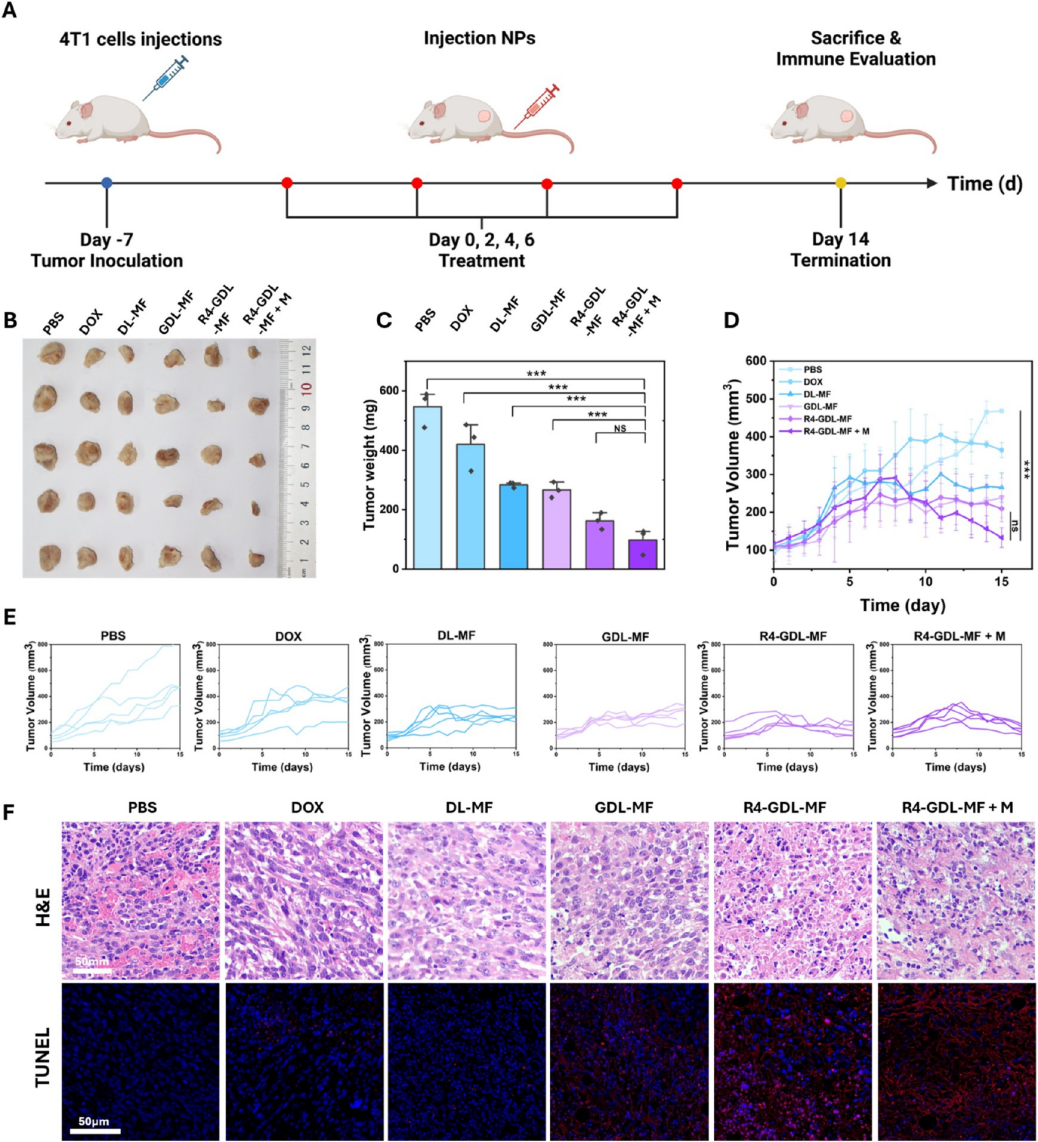
*In vivo* antitumor efficacy. (A) Schematic diagram of *in vivo* therapy (MF, DL-MF, GDL-MF, R4-GDL-MF, and R4-GDL-MF + M). (B) Tumor images after treatments. (C) Average tumor weights (*n* = 3). (D) Average and (E) individual tumor volume (*n* = 5). (F) H&E and TUNEL staining.

### *In Vivo* Immune Evaluation

Next, we investigated the immune response triggered by R4-GDL-MF *in vivo*, building upon our *in vitro* findings that RM-GDL-MF effectively induces ICD. Consistent with these *in vitro* results, R4-GDL-MF treatment significantly elevated the secretion of proinflammatory cytokines TNF-α and IL-6 (Figures S18 and S19) and induced macrophage polarization toward an antitumor M1 phenotype *in vivo*. To evaluate immune activation, tumors were harvested from 4T1 tumor-bearing mice after 14 days of R4-GDL-MF treatment and analyzed by flow cytometry (Figure 8A). R4-GDL-MF treatment markedly increased the proportion of CD8^+^ T cells to 6.65%, approximately 3-fold higher than in the control group (Figure 8B,C). Activated CD4^+^ helper T cells were also elevated in the R4-GDL-MF group compared to controls (Figure S20).

**Figure 8:**
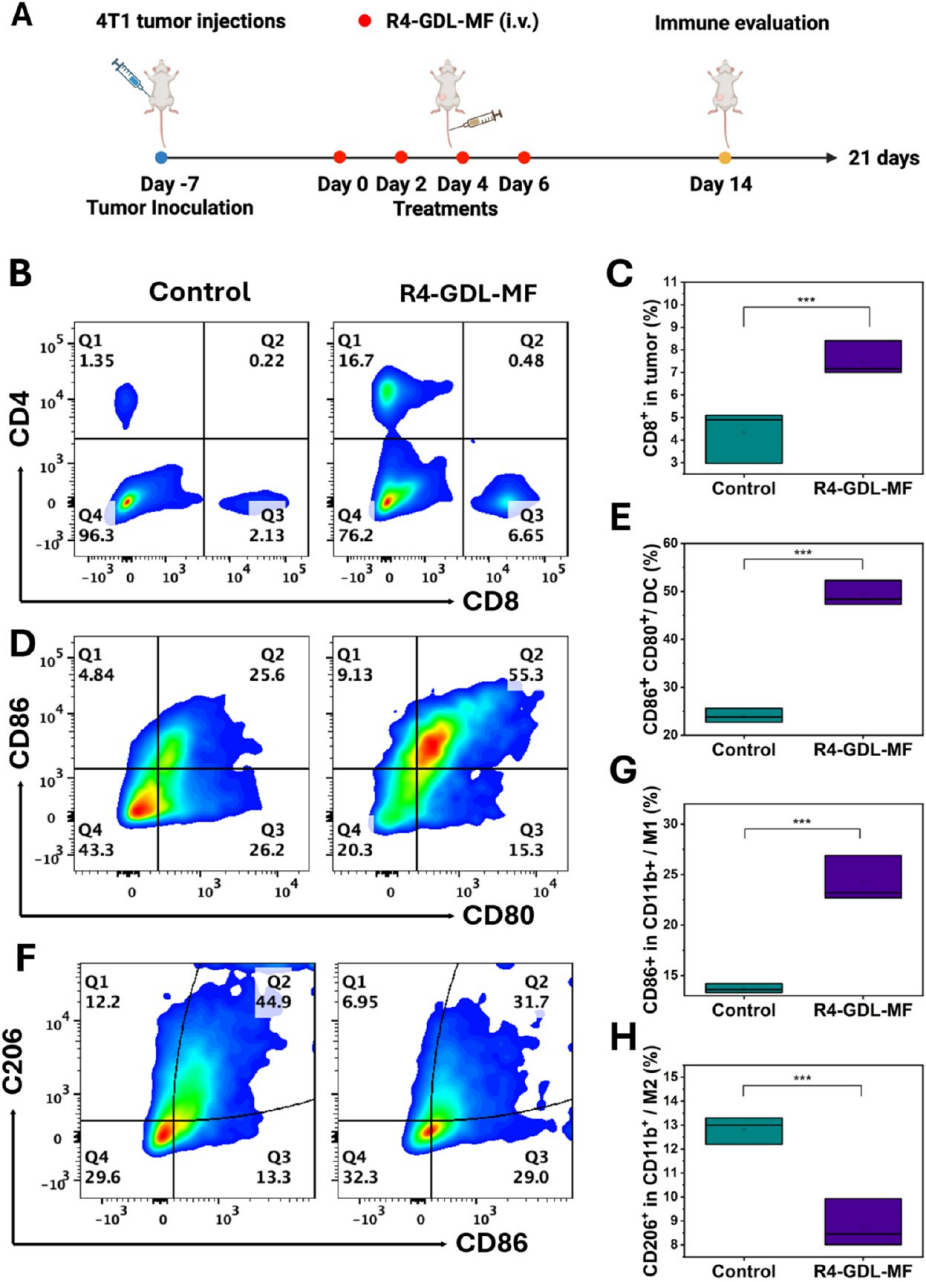
*In vivo* immune evaluation. (A) Schematic diagram of immune evaluation. (B) Flow cytometry plots of intratumoral CD8^+^ T cells and (C) their quantification. (D) Flow cytometry plots of mature dendritic cells (CD86^+^CD80^+^) and (E) their quantification. (F) Flow cytometry plots of M1 (CD86^+^ in CD11b^+^) and M2 (CD206^+^ in CD11b^+^) macrophages, with quantification shown in (G) for M1 and (H) for M2.

In addition, R4-GDL-MF treatment markedly enhanced dendritic cell (DC) maturation, as evidenced by the elevated CD80^+^ and CD86^+^ populations. Mature DCs accounted for 55.3% of the total population, compared to only 25.6% in the control group (Figure 8D,E), highlighting the potent immune-stimulating capability of the treatment. Furthermore, macrophage polarization analysis revealed that R4-GDL-MF induced the highest proportion of M1 macrophages (∼29%), about 2.5-fold higher, as compared to the control group, while reducing the M2 phenotype to ∼6.95%, nearly half of the control level (Figure 8F–H). Besides, immunofluorescence staining of tumor sections also revealed pronounced red fluorescence (CD86) in GDL-MF-, R4-GDL-MF-, and R4-GDL-MF + M-treated groups (Figure S21), highlighting substantial M1 macrophage polarization. Conversely, control and DOX-treated groups predominantly exhibited green fluorescence (CD206), consistent with M2 macrophage dominance. Collectively, these results indicate that R4-GDL-MF not only triggers ICD but also reshapes the immunosuppressive tumor microenvironment into a proinflammatory state, thereby enhancing antitumor immunity.

Finally, we assessed the biosafety of R4-GDL-MF. Blood biochemical analysis, including measurements of alanine aminotransferase (ALT), aspartate aminotransferase (AST), creatine kinase (CK), blood urea nitrogen (BUN), and creatinine (CREA), showed no significant differences between the treatment groups (Figure S22). Histological analysis of collected organs via H&E staining also revealed no detectable abnormalities or tissue damage (Figure S23). These results suggest that the side effects of R4-GDL-MF are negligible, further supporting its potential as a safe and effective cancer therapy.

## Conclusions

We successfully developed a multifunctional hybrid membrane-coated mesoporous silica-iron oxide nanoplatform loaded with DOX, l-arg, and Gox. By integrating cancer cell membranes for precise tumor targeting with RBC membranes for effective immune evasion, our design achieves excellent specificity and prolonged circulation, enabling RM/R4-GDL-MF to exhibit an excellent tumor-targeting capability in both *in vitro* and *in vivo* experiments. The synergistic release of DOX, ROS, and NO orchestrates a potent chemotherapeutic and immunotherapeutic effect that robustly activates antitumor immunity. The cascade reaction between GOx and l-arg within RM/R4-GDL-MF generated substantial amounts of NO and H_2_O_2_, which not only facilitated autonomous motion for improved tumor penetration but also promoted macrophage polarization toward the immunostimulatory M1 phenotype. Moreover, RM-GDL-MF was found to effectively induce ICD, as evidenced by elevated levels of CRT exposure, ATP secretion, and HMGB1 release. These DAMPs further enhanced the maturation of dendritic cells and T cell activation, amplifying the systemic antitumor immune response. Importantly, the l-arg–driven nanomotor, further guided by an external magnetic field via the Fe_3_O_4_ core, ensures deep tumor penetration and enhanced intratumoral distribution, overcoming conventional delivery barriers. Collectively, these integrated functionalities establish a robust platform for simultaneous chemoimmunotherapy and precise tumor targeting, paving the way for advanced therapeutic strategies against triple-negative breast cancer.

## Methods

### Materials and Reagents

Iron oxide nanoparticle was purchased from Macklin (China). Tetraethyl orthosilicate (TEOS) and (3-aminopropyl)­triethoxysilane (APTES) were obtained from Sigma-Aldrich (USA). The hydrogen peroxide kit and nitric oxide kit were obtained from Beyotime (China). Succinic anhydride was obtained from Aladdin (China). Doxorubicin (DOX) and glucose oxidase (GOX) were purchased from Macklin (China). l-arg was obtained from Sigma-Aldrich (USA). Mouse red blood cell was obtained from Bioivt (USA). The protein membrane extraction kit was purchased from Beyotime (China). SDS loading buffer, PMSF, and RIPA lysis solution were purchased from Beyotime (China). Dulbecco’s modified Eagle’s medium (DMEM), fetal bovine serum (FBS), penicillin, and streptomycin were purchased from Thermo Fisher Scientific Ltd. MTT, Hoechst 33342, DCFH-DA kit, DAF-FM-DA kit, Calcein-AM/PI kit, JC-1, and Apoptosis kit were obtained from Beyotime Biotech Inc. Anti-CRT was obtained from Servicebio. Anti-HMGB1 was purchased from Biolegend. ATP assay was obtained from Beyotime. The following fluorescent antibodies were purchased from BioLegend (USA): PE-CD8a, APC-CD4, APC-CD80, PE-CD86, APC-CD206, PE antimouse, FITC antimouse/human CD11b antibody, and FITC antimouse CD45 antibody. The LIVE/DEAD Fixable Blue Dead Cell Stain Kit (for UV excitation, ex: 350 nm, em: 450 nm) was purchased from Thermo Fisher Scientific Ltd.

### Characterization Methods

The transmission electron microscopy (TEM) image was obtained by TEM-FEI TS12 (G20). Hydrodynamic diameter and ζ-potential were measured by dynamic light scattering (DLS) of a Malvern Instrument Zetasizer ZS90 (G14). Absorbance of different assays (MTT, DOX) was measured by a Microplate reader (Multiskan FC, Thermo Scientific, USA). Motion behavior of Nanoparticles was measured by Nanoparticle Tracking Analysis (Malvern Panalytical, United Kingdom). Confocal images were taken by a confocal laser scanning microscope (Leica TCS SP8). Flow cytometry was done by a BD Symphony (A5.2 SORP High-parameter). The ELISA tests of TNF-α and IL-6 were done by a commercial laboratory (Wuhan Servicebio technology CO LTD). The biochemical index for liver and kidney, including alanine aminotransferase (ALT), aspartate aminotransferase (AST), phosphocreatine kinase (CK), creatinine (CRE) and blood urea nitrogen (BUN), were measured by commercial laboratory (Wuhan Servicebio Technology CO LTD). TGA analysis was performed using a PerkinElmer Pyris 1 TGA.

### Synthesis of Mesoporous Silica-Coated Iron Oxide Nanoparticles (MF)

Silica-coated iron oxide (Si–Fe_3_O_4_) was prepared by the sol–gel method. First, 1 mL of iron oxide (33 mg) was added to the mixture of 29 mL of DI water, 27.5 mL of EtOH, and 5.35 mL of NH_3_ (28 wt %) and homogenized for 30 min. Then, 1 mL TEOS in 30 mL EtOH was added dropwise into the mixture. After reacting for 4 h, the obtained brown suspension was washed with EtOH three times. The final suspension was redispersed in 10 mL EtOH. Mesoporous silica-coated iron oxide nanoparticle was synthesized according to literature.^16^ Briefly, silica-coated iron oxide was dispersed in a mixture of 0.3 g cetyltrimethylammonium bromide (CTAB), 60 mL of ethanol, 80 mL of deionized water, and 0.5 mL of 28% ammonia aqueous solution and sonicated for 30 min. Then, 0.2 mL of TEOS was added into the mixture and continuously stirred for 6 h at room temperature. The final nanoparticle was washed with ethanol three times and dispersed in 1 mL of ethanol.

### Preparation for NH_2_-MF

50 mg portion of MF was dispersed in 50 mL of toluene in a 100 mL two-neck round-bottom flask. One mL of APTES was added, and the mixture was refluxed at 80 °C overnight. The final product was washed with ethanol three times and dispersed in 10 mL of ethanol.

### Preparation of COOH-MF

50 mg of NH_2_-MF was dispersed in 50 mL of DMF in a 100 mL two-neck round-bottom flask. 0.25 mg of succinic anhydride was added, and the mixture was stirred overnight protected by nitrogen. The final product was washed with ethanol three times and dispersed in 10 mL ethanol.

### Loading of l-arg and DOX into MF (DL-MF)

DL-MF was prepared by loading l-arg and DOX into COOH-MF. Briefly, 20 mg of l-arg and 2 mg of DOX were dissolved in 1 mL of PBS (1×) and added into 1 mL of well-dispersed MF (4 mg/mL). The mixture was sonicated until homogeneous and vortexed overnight protecting from the night. Then, the mixture was washed with PBS three times and dispersed in 1 mL of PBS.

### Preparation of RBC Membrane and Cancer Cell Membrane

For the extraction of the RBC membrane, commercial red blood cells were used for better purity and quality of the RBC membrane.^17^ Red blood cells were dispersed in the hypotonic solution (0.25× PBS) and immersed into an ice bath for 20 min. The RBC solution was centrifuged at 13,000 rpm at 4 °C to wash away the hemoglobin until the supernatant became colorless. The pink pellet was collected and redispersed in 1× PBS and stored at −80 °C for further use. For the extraction of the cancer cell (MDA-MB-231/4T1) membrane, a commercial membrane protein extraction kit was used. The preparation of the cancer cell membrane was done under the manufacturer’s instructions. Briefly, the cells were incubated in DMEM medium with 10% FBS and 1% penicillin/streptomycin at 37 °C with 5% CO_2_ for 48h. The cells were harvested when the number of cells reached 5 × 10^6^. Cells were washed with 1× PBS several times and centrifuged at 1000 rpm for a minute. The cells were resuspended in 1 mL of membrane protein extraction reagent A containing PMSF (1 mM). The mixture was incubated in an ice bath for 15 min and then centrifuged at 3000 rpm and 4 °C for 15 min. The supernatant was further centrifuged at 13,000 rpm at 4 °C for 30 min to obtain the membrane, which was lyophilized and stored at −80 °C for further use.

### Loading of GOx into DL-MF

DL-MF (0.25 mL, 0.4 mg/mL) and GOx (0.25 mL, 20 μg/mL) were mixed and sonicated for 20 min at room temperature. RBC (0.25 mL, 0.4 mg/mL) were mixed with MDA (0.25 mL, 0.4 mg/mL) and sonicated for 10 min in room temperature. [RBC-MDA] were mixed with a GDL-MF mixture and sonicated for 10 min at room temperature. The resulting solution was centrifuged at 10,000 rpm to remove excess cell membrane and GOX.

### H_2_O_2_ Quantification of RM-GDL-MF

A hydrogen peroxide kit was used to measure the amount of H_2_O_2_ released after the addition of glucose solution into RM-GDL-MF. After the addition of 5 mM glucose solution into different concentrations of RM-GDL-MF solution (0, 200, 400, and 800 μg/mL), the reaction mixture was incubated in a 37 °C water bath. The quantification method was conducted according to the manufacturer’s instructions, 50 μL of supernatant was extracted at predetermined time points (1, 3, 6, 9, 12, 24 h) and added to a 96-well plate with H_2_O_2_ kit’s reagent. The standard curve was developed using hydrogen peroxide provided in the kit (0, 1, 5, 10, 20, 50, 100 μM). The absorbance was read using a microplate reader at 620 nm.

### NO Quantification of RM-GDL-MF

A nitric oxide assay kit was used to measure the amount of NO release after the addition of glucose solution into RM-GDL-MF. After the addition of 5 mM glucose solution into different concentrations of RM-GDL-MF solution (0, 200, 400, and 800 μg/mL), reaction mixtures were incubated in 37 °C water bath. The quantification method was conducted according to the manufacturer’s instructions; 50 μL of supernatant was extracted at a predetermined time point (1, 3, 6, 9, 12, 24 h) and added into a 96-well plate with Griess reagent. The standard curve was developed using hydrogen peroxide provided in the kit (0, 0.25, 0.5, 0.75, 1, and 2.5 μM) The absorbance was read using a microplate reader at 560 nm.

### DOX Release of RM-GDL-MF

The standard curve of DOX was developed by preparing DOX stock solutions at different concentrations (0, 10, 50, 200, and 500 μg/mL). After addition of 5 mM glucose solution into different concentrations of RM-GDL-MF solution (0, 200, 400, and 800 μg/mL), reaction mixtures were incubated in a 37 °C water bath. 50 μL of supernatant was extracted at a predetermined time point (1, 3, 6, 9, 12, 24 h) and added into a 96-well plate. 100 μL DI water was added into the supernatant. The absorbance was read by microplate reader at 480 nm.

### Motion Behavior of RM-GDL-MF

The motion behavior of RM-GDL-MF is characterized by NTA analysis. The autonomous motion of RM-GDL-MF in 0 and 500 μM glucose solutions is recorded and used to calculate the Mean Square Displacement (MSD). The *x* and *y* coordinates of the RM-GDL-MF nanoparticles (*n* = 20) were tracked and recorded for 30 s. The MSD can be calculated using the following equation.

$$\mathrm{MSD}\left(t\right)=\left\langle {\left(\Delta x\right)}^{2}+{\left(\Delta y\right)}^{2}\right\rangle =\left\langle {\left(\Delta r\right)}^{2}\right\rangle =\frac{1}{N}\sum_{i=1}^{N}\left({\left(r_{i}\left(t\right)-r_{i}\left(0\right)\right)}^{2}\right)$$

Where *N* = the total number of particles, *r_i_* (*t*) is the position of particle at time *t*, and *r_i_* (0) is the position of the particle at the initial position (time = 0). The diffusion coefficient is extracted from the NTA analysis.

### Characterization of RBC-MDA Hybrid Membrane Coated on MF

The successful coating of the hybrid membrane of RBC-MDA was confirmed using CLSM. RBC and MDA membranes were prelabeled with Dil and DiO, respectively. Briefly, 150 μL of a mixture of RBC membrane (1 mg/mL) was premixed with 15 μL of DiO dye (1 mg/mL, in DMSO) for 2 h in the dark, respectively.

### Characterization of Membrane Protein by SDS-PAGE

The PMSF was added to the RIPA lysate until it reached 1 mM. RM-GDL-MF was lysed with PMSF lysate and centrifuged at 13,500 rpm for 5 min. The supernatants were taken for SDS-PAGE. All samples were mixed with SDS-PAGE and heated in 100 °C boiling water for 10 min. Samples with an equivalent amount of protein (40 μg/well) were loaded on SDS-PAGE gel, loading the protein ladder (5–10 μL) on the first lane. The SDS-PAGE gel was run at 120 V for 2 h. After electrophoresis, the gel was stained with Coomassie solution.

### Thermogravimetric Analysis (TGA)

Approximately 5.0 mg of each nanoparticle sample (MF, DL-MF, GDL-MF, and R4-GDL-MF) in dried powder form was weighed and placed in a clean platinum crucible. The samples were loaded into the thermogravimetric analyzer and heated from 30 to 800 °C at a constant rate of 10 °C min^–1^ under a nitrogen atmosphere (flow rate: 50 mL min^–1^). The mass change was continuously recorded throughout the heating process. The percentage weight loss was calculated to determine the amount of organic matter (drug, enzyme, and membrane coating) in each formulation, and the residual weight at 800 °C was attributed to the inorganic MF core.

### Cell Culture

Human breast cancer MDA-MB-231 cell line, Murine breast cancer 4T1 cell line, mouse fibroblast L929 cell line, and macrophage raw264.7 cell line were cultured in Dulbecco’s Modified Eagle Medium (DMEM) supplemented with 10% fetal bovine serum (FBS), 1% penicillin, and streptomycin at 37 °C in a humid atmosphere with 5% CO_2_.

### Cellular Uptake

MDA-MB-231, 4T1, and raw264.7 cells were used to evaluate the cellular uptake of different NPs. Cells were seeded in an 8-well confocal chamber slide at a density of 0.2 × 10^5^ per well overnight. Rhodamine b-labeled NPs (20 μg/mL) were redispersed in cell culture medium and incubated with different cells for 4 h. Cells were washed with PBS three times and stained with Hoechst 33342 (1 μg/mL) for 10 min. The fluorescence intensity of each group was detected by confocal microscopy.

### Live/Dead Cell Imaging

MDA-MB-231 cells were seeded in an 8-well confocal chamber slide at a density of 0.2 × 10^5^ per well overnight. Then, MDA-MB-231 cells were incubated with different NPs (200 μg/mL) for 24 h. The cells were then stained with Calcein-AM/PI for 30 min. The fluorescence intensity of each group was detected by confocal microscopy.

### Cytotoxicity Assay

MDA-MB-231 cells and L929 cells were seeded in a 96-well plate at a density of 0.2 × 10^5^ per well overnight. MDA-MB-231 cells and L929 cells were incubated with different NPs with different concentrations (0, 10, 50, 100, 200 μg/mL) for 24 h. The survival rate of each group of cells was detected by MTT assay.

### Apoptosis

MDA-MB-231 cells were seeded in six-well plates at a density of 0.3 × 10^6^ cells per well overnight. MDA-MB-231 cells were incubated with different NPs (200 μg/mL) for 24 h. Cells were digested and collected from 6-well plates. Cells were then stained with a mixture of Annexin V-FITC and PI for 30 min and analyzed by flow cytometry.

### DCF Assay (ROS)

MDA-MB-231 cells were seeded in an 8-well confocal chamber slide at a density of 0.2 × 10^5^ per well overnight. Then, MDA-MB-231 cells were incubated with different NPs (20 μg/mL) for 4 h. The cells were then stained with DCFH-DA for 20 min. Cells were washed with PBS three times and stained with Hoechst 33342 (1 μg/mL) for 10 min. The fluorescence intensity of each group was detected by confocal microscopy.

### DAF-FM Assay (NO)

MDA-MB-231 cells were seeded in an 8-well confocal chamber slide at a density of 0.2 × 10^5^ per well overnight. Then, MDA-MB-231 cells were incubated with different NPs (20 μg/mL) for 4 h. The cells were then stained with DAF-FM-DA for 20 min. Cells were washed with PBS three times and stained with Hoechst 33342 (1 μg/mL) for 10 min. The fluorescence intensity of each group was detected by confocal microscopy.

### JC-1 Assay

MDA-MB-231 cells were seeded in an 8-well confocal chamber slide at a density of 0.2 × 10^5^ per well overnight. MDA-MB-231 cells were incubated with different NPs (20 μg/mL) for 4 h. The cells were then stained with JC-1 dye in a JC-1 buffer for 30 min. The cells were washed with JC-1 buffer three times. The fluorescence intensity of each group was detected by confocal microscopy.

### HMGB1 and CRT Staining

MDA-MB-231 cells were seeded in an 8-well confocal chamber slide at a density of 0.2 × 10^5^ per well and were incubated with different NPs (200 μg/mL) for 24 h. The cells were then fixed and permeated and incubated with HMBG1 and CRT antibody overnight. Cells were washed with PBS three times and stained with DAPI for 10 min. The fluorescence intensity of each group was measured by confocal microscopy.

### ATP Assay

MDA-MB-231 cells were seeded in a 96-well plate at a density of 0.2 × 10^5^ per well and were incubated with NPs (200 μg/mL) overnight. Supernatants were extracted for analysis. The ATP levels of different treatments were measured according to the manufacturers’ instructions.

### Hemolysis Assay

For biosafety evaluation, to measure the hemocompatibility, a hemolysis assay was carried out. Briefly, fresh red blood cells (RBCs) were first isolated from the whole blood of healthy BALB/c mice with a centrifuge (4000 rpm, 10 min) and then washed with PBS 5 times (until the supernatant was clear). The NPs were diluted to 0, 5, 10, 20, 50, 100, 200, 400, or 800 μg/mL with PBS to reach a final volume of 200 μL. PBS and deionized water were set as the negative group and positive group, respectively. After that, fresh RBCs (10 μL) were added to each of the above nanogel suspensions. The resultant RBC suspensions were incubated at room temperature for 2 h and then centrifuged at 4000 rpm for 5 min to collect their supernatants. The supernatants were put in a 96-well plate to measure the absorbance at 570 nm using a microplate reader (Multiskan FC, Thermo Scientific, USA). The percentage of hemolysis was calculated according to the following equation

$$h\mathrm{emolysis}\left(\%\right)=\frac{{a\mathrm{bsorbance}}_{\mathrm{sample}}-{a\mathrm{bsorbance}}_{\mathrm{negativecontrol}}}{{a\mathrm{bsorbance}}_{p\mathrm{ositivecontrol}}-{a\mathrm{bsorbance}}_{\mathrm{negativecontrol}}}\times 100\%$$

### Biodistribution

*In vivo* biodistribution was examined through an IVIS imaging system. BALB/c mice (female, 4–6 weeks old, *n* = 3) established 4T1 xenograft tumor model by subcutaneously inoculating 4T1 cells (5 × 10^6^, 100 μL) in the flank position. When the tumor size reached 100 mm^3^, the mice were divided into three groups. R4-GDL-MF were prelabeled with DiR fluorescence dye. A tail vein injection was performed of (1) R4-GDL-MF and (2) R4-GDL-MF + Magnet. The major organs were imaged using an IVIS spectrum imaging system at desired time points (0, 4, 8, 12, 24 h). The organs and cancer tissues were collected from the sacrificed mice at 24 h and observed using the IVIS.

### Therapeutic Evaluation *In Vivo*

The BALB/c mice (female, 4–6 weeks old) with subcutaneous 4T1 tumor xenografts (5 × 10^6^, 100 μL/mice, in the right flank) were divided into 6 groups according to the different treatments (*n* = 5 per group): (1) PBS, (2) DOX in normal saline (4 mg/kg), (3) DL-MF, (4) GDL-MF, (5) R4-GDL-MF, (6) R4-GDL-MF + Magnet. After 7–10 days of inoculation, when the tumor size reached 100 mm, all the samples were intravenously injected into mice via the tail vein (MF 10 mg/kg). A small magnet was attached to the tumor after injection (group 6). The treatments were conducted every 2 days 4 times. The tumor volume and mouse body weight were measured every other day. Tumor volume was calculated according to the following formula: (width^2^ × length × 0.5). After 14 days, all the mice were euthanized, blood was extracted for blood biochemical analysis, and the major organs and tumors were collected for H&E staining and TUNEL staining. The tumor and major organs are dipped into 4% paraformaldehyde for storage and further analysis.

### Immune Evaluation *In Vivo*

The tumors harvested after 14 days were sliced. Part of the tumor was fixed with 4% paraformaldehyde for histological sectioning. The slices were stained with anti-206-PE, anti-86-APC, and DAPI for immunofluorescence image. Serum samples were collected from the mice after treatments. TNF-α and IL-6 were quantified using ELISA kits. The concentration of cytokines can be determined by using a microplate reader according to the manufacturer’s instruction. For flow cytometry analysis, the 4T1 tumor-bearing mice and treatment regimens were conducted as described in the tumor suppression experiments. Tumors were cut into small fragments (2 and 4 mm) and digested in an enzyme mixture containing collagenase IV (1 mg/mL), hyaluronidase (0.1 mg/mL), and DNase I (100 U/mL) at 37 °C for 45 min. The digested tissue was further dissociated into a single-cell suspension, filtered, and washed with cold PBS. Cells were then incubated with the following fluorophore-conjugated antibodies for surface staining: APC-CD4, PE-CD8, APC-CD80, PE-CD86, FITC-CD11b, and APC-CD206. Samples were analyzed by flow cytometry to quantify the populations of T cells, dendritic cells, and macrophage subtypes.

### Biosafety

After 14 days of treatment, mice were sacrificed. The major organs (heart, liver, spleen, lung and kidney) and blood were collected, weighted, and fixed with 4% paraformaldehyde for histological sectioning. Part of organs were sliced and stained with H&E to evaluate the tissue damage. For blood biochemical analysis, serum was collected to measure the levels of alanine aminotransferase (ALT), aspartate aminotransferase (AST), phosphocreatine kinase (CK), creatinine (CRE), and blood urea nitrogen (BUN).

### Statistical Analysis

The data are demonstrated as mean ± standard deviation (SD), and the statistical significance of the data was evaluated by the one-way analysis of variance (ANOVA) method. Data are expressed as mean ± s.d., *n* = 3 or 5, *p < 0.05, **p < 0.01, ***p < 0.001, respectively.

## Supplementary Material







## Notes

Size distribution of mesoporous iron oxide nanoparticles (MF) (Figure S1); ζ-potentials of MF, NH_2_-MF, and COOH-MF (Figure S2); FTIR spectra of Si–Fe_3_O_4_, MF, NH_2_-MF, and COOH-MF (Figure S3); TEM image of DL-MF and GDL-MF (Figure S4); FTIR spectra of DOX, l-arg, GOX, MF, DL-MF, GDL-MF, and RM-GDL-MF (Figure S5); calibration curves of l-arg (Figure S6) and DOX (Figure S7) for the quantification of unloaded l-arg in the supernatant; confocal images of cellular uptake of DiI-labeled MF and DiD-labeled hybrid cell membranes (Figure S8); thermogravimetric analyses (TGA) of MF, DL-MF, GDL-MF, and R4-GDL-MF (Figure S9); DOX release of DL-MF with and without the coating of hybrid membranes at pH 5.5 and pH 7.4 (Figure S10); hemolysis assay of different NPs at various concentrations. (Figure S11); *in vitro* cytotoxicity of RM-GDL-MF on L929 cells (Figure S12); ATP release of RM-GDL-MF in MDA-MB-231 cells (Figure S13); TEM images of R4-GDL-MF (Figure S14); *in vitro* cytotoxicity of R4-GDL-MF on 4T1 cells (Figure S15); MF in EtOH before and after attaching to a magnet (Figure S16); body weight record of mice in different treatment groups (Figure S17); serum TNF-α (Figure S18) level and serum IL-6 level (Figure S19) of mice after 14 days of treatment with and without R4-GGDL-MF; quantification of intratumoral CD4^+^ T cells by flow cytometry analysis (Figure S20); immunofluorescence of CD86 and CD206 in tumor slice (Figure S21); and blood biochemistry. Liver and kidney index upon treatment (1: PBS, 2: DOX, 3: DL-MF, 4: GDL-MF, 5: R4-GDL-MF, 6: R4-GDL-MF + M) (Figure S22); and H&E staining of organs (heart, kidneys, liver, lungs, and spleen) of different treated groups (Figure S23) (PDF)
Particles moving in 0 μM glucose solutions (Video S1) (AVI)
Particles moving in 500 μM glucose solutions (Video S2) (AVI)
