# Supplementary material for: Multifunctional Hybrid Membrane-Coated Nanomotors for Magnetically Guided, Cascade-Activated Chemoimmunotherapy for Triple-Negative Breast Cancer
Source: ACS Appl Mater Interfaces. 2025 Sep 4;17(37):51643–58. doi: 10.1021/acsami.5c10644 (PMC12447376; doi:10.1021/acsami.5c10644)
Supplement: Supplementary file 1 [file am5c10644_si_001.pdf]

Supporting Information

Multifunctional Hybrid Membrane-Coated Nanomotors for Magnetically Guided, Cascade-Activated Chemoimmunotherapy of Triple-Negative Breast Cancer

‡Man Lung Lee[a], ‡Li Wang[b], Jack Chun Hin Chen[c], Ellen Ngar-Yun Poon[c,d], Dinggeng He[b,], Hung-Wing Li[a,*]*

[a]Department of Chemistry, The Chinese University of Hong Kong, Shatin, Hong Kong SAR, China

[b]College of Life Science, Hunan Normal University, Changsha 410081, P. R. China

[c]School of Biomedical Sciences, The Chinese University of Hong Kong, Shatin, Hong Kong SAR, China

[d]Hong Kong Hub of Paediatric Excellence (HK HOPE), The Chinese University of Hong Kong, Shatin, Hong Kong SAR, China

‡M.L. Lee and L. Wang contributed equally

* Correspondence: Dinggeng He hedinggeng@hnu.edu.cn and Hung-Wing Li hungwingli@cuhk.edu.hk

Supplementary Figures

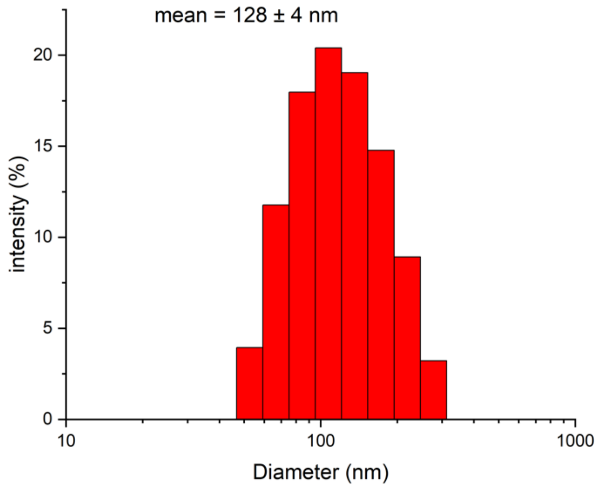

Figure S1. Size distribution of mesoporous iron oxide nanoparticles (MF).

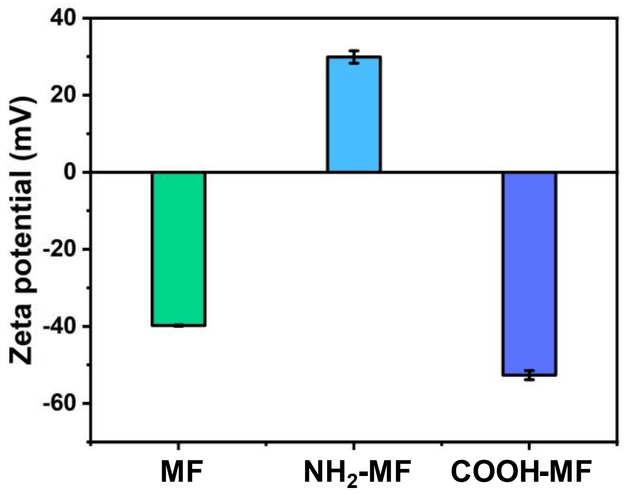

Figure S2. Zeta potential of MF, NH_2-MF and COOH-MF.

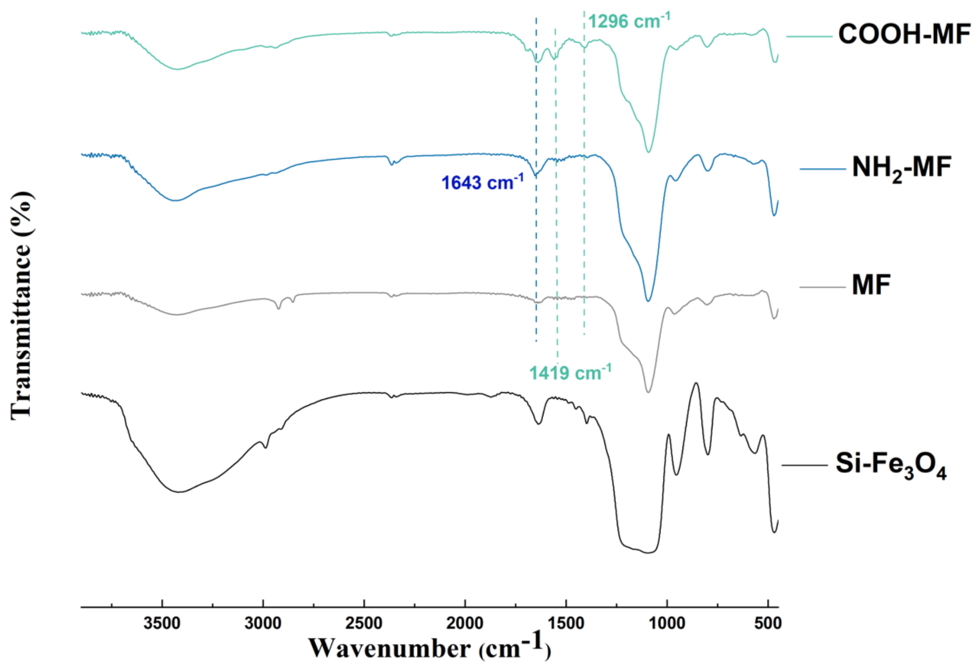

Figure S3. FTIR spectra of Si-Fe_3O_4, MF, NH_2-MF and COOH-MF.

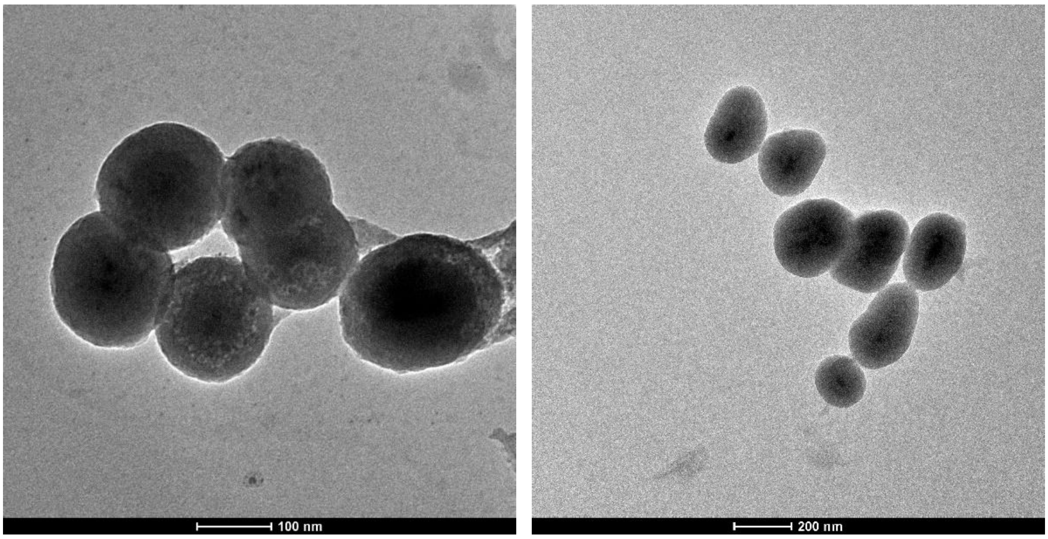

Figure S4. TEM images of DL-MF (left), and GDL-MF (right).

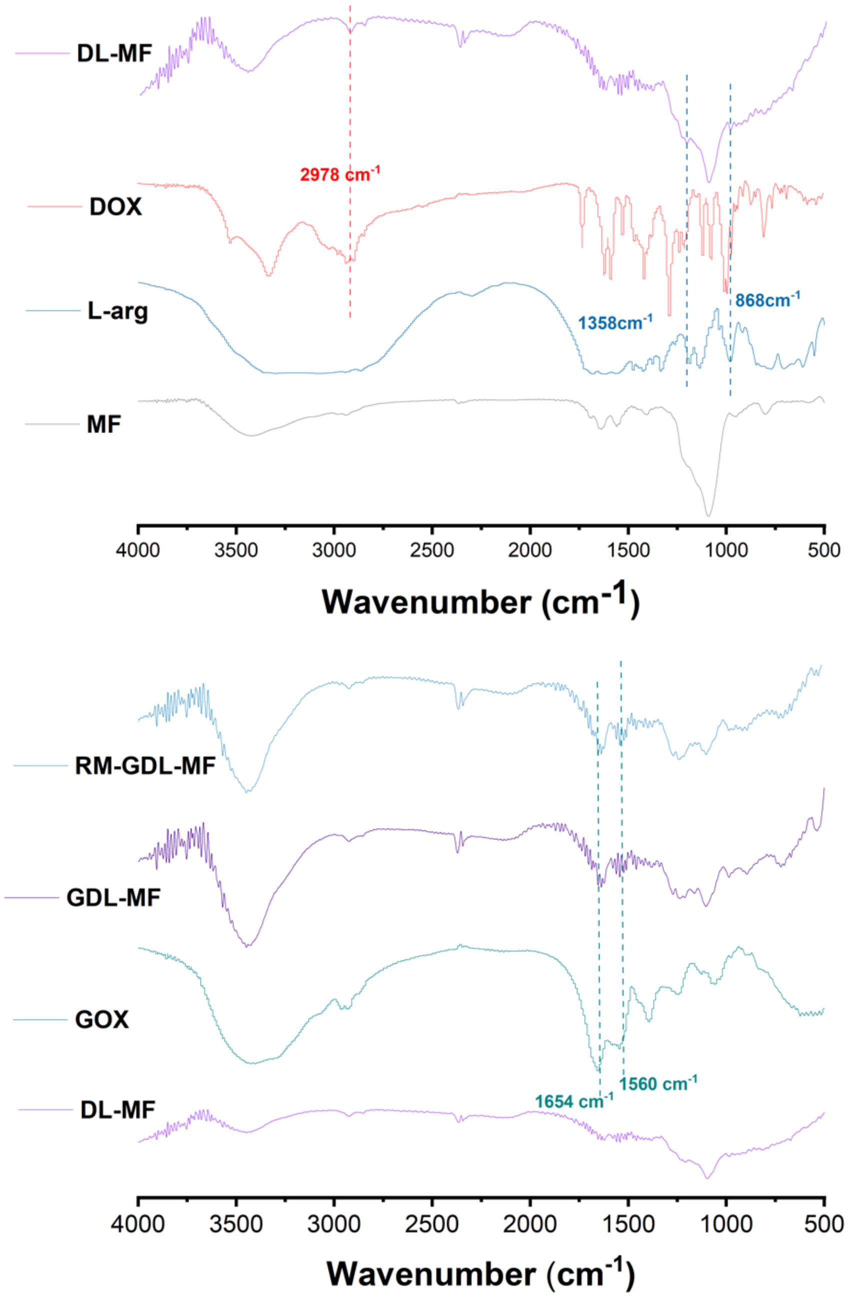

Figure S5. FTIR spectra of DOX, L-arg, GOX, MF, DL-MF, GDL-MF and RM-GDL-MF.

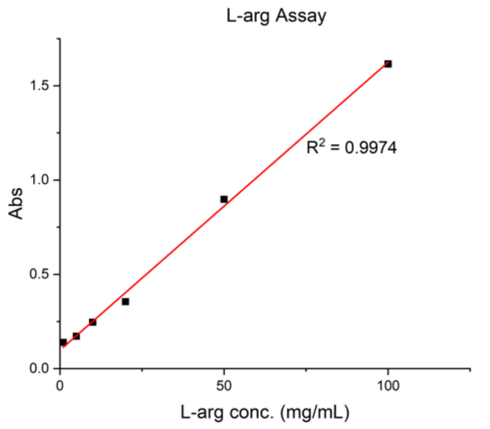

Figure S6. Calibration curve of L-arg at 559 nm for the quantification of unloaded L-arg in the supernatant.

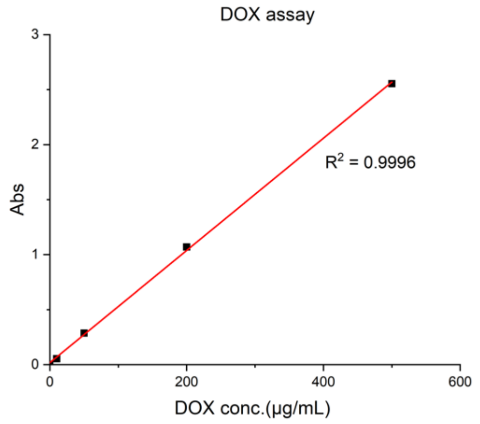

Figure S7. Calibration curve of DOX at 480 nm for the quantification of unloaded DOX in the supernatant.

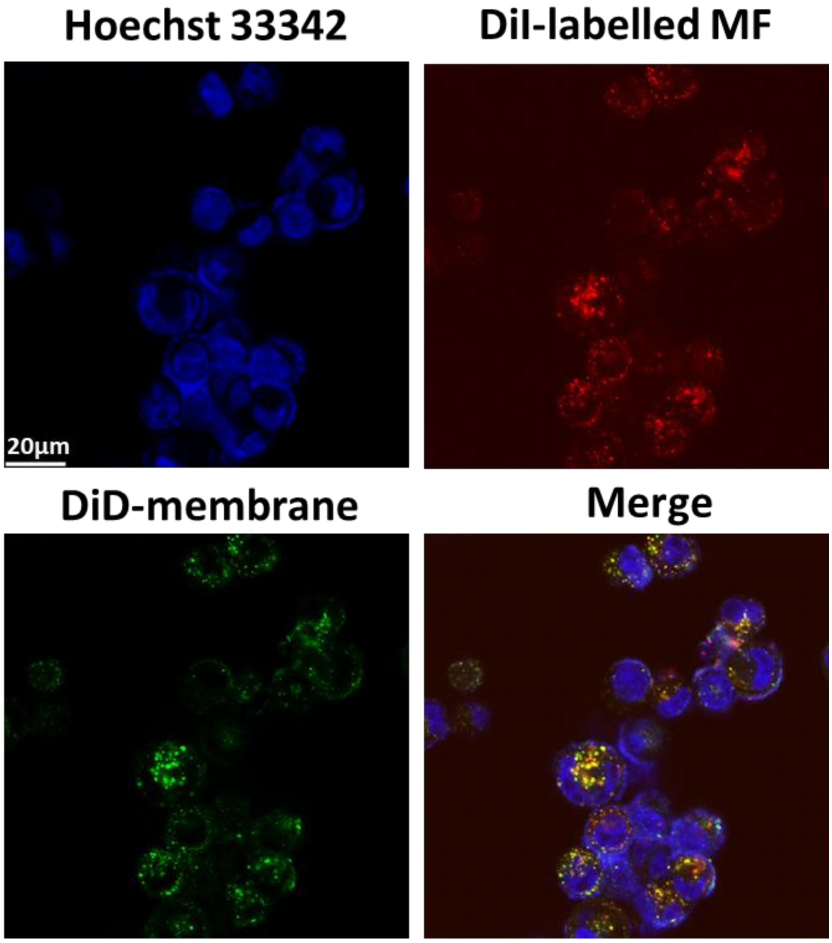

Figure S8. Confocal images of cellular uptake of DiI-labelled MF and DiD-labelled hybrid cell membranes.

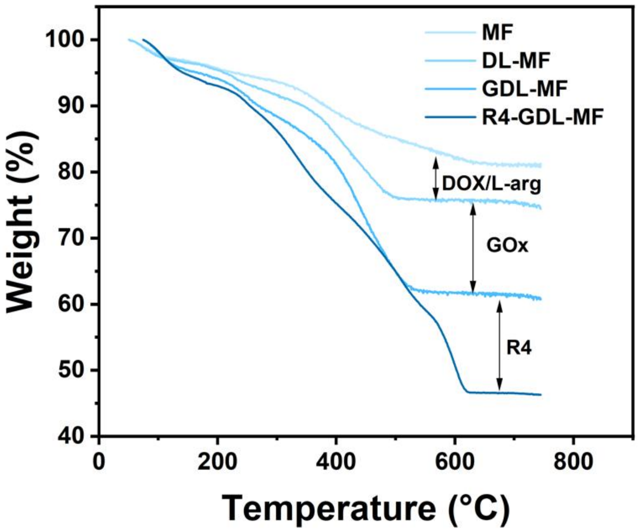

Figure S9. Thermogravimetric analyses (TGA) of MF, DL-MF, GDL-MF and R4-GDL-MF.

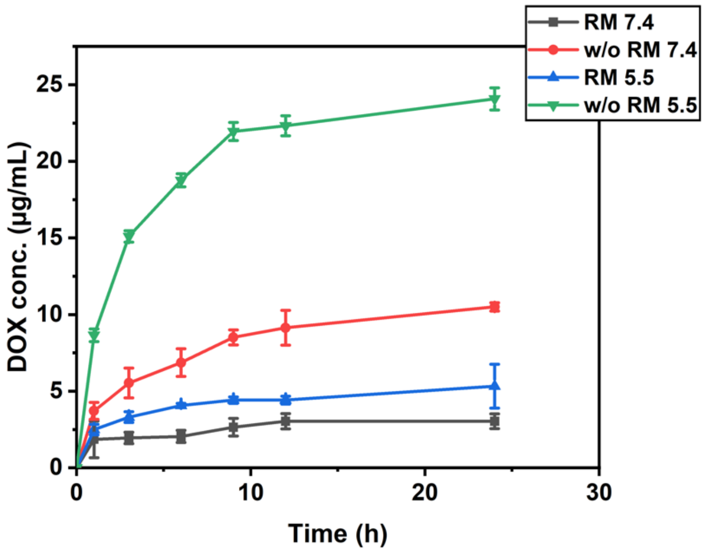

Figure S10. DOX release of DL-MF with and without the coating of hybrid membranes at pH 5.5 and pH 7.4.

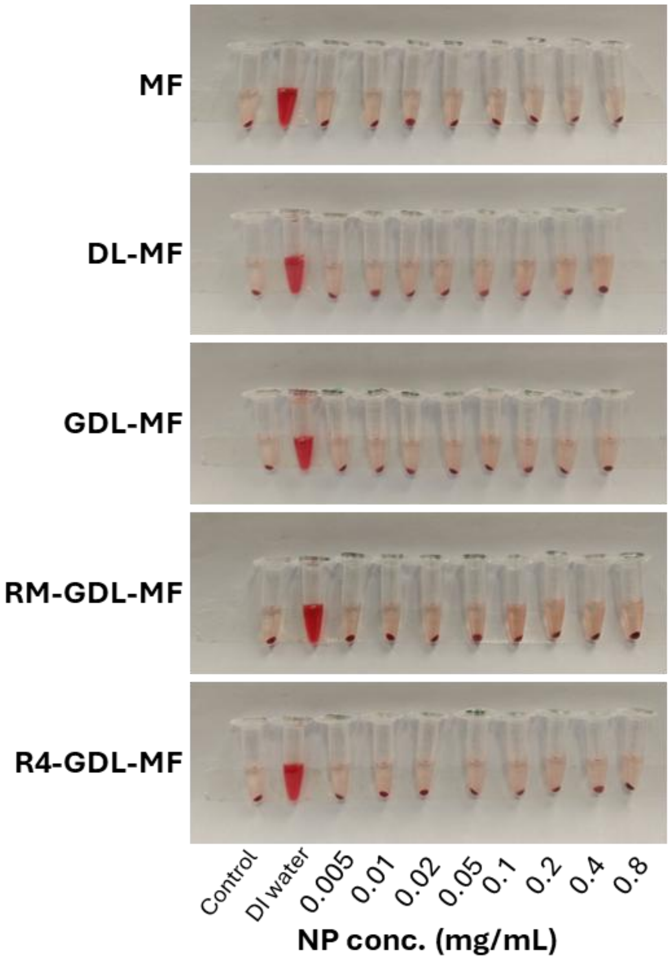

Figure S11. Hemolysis assay of different NPs at various concentrations.

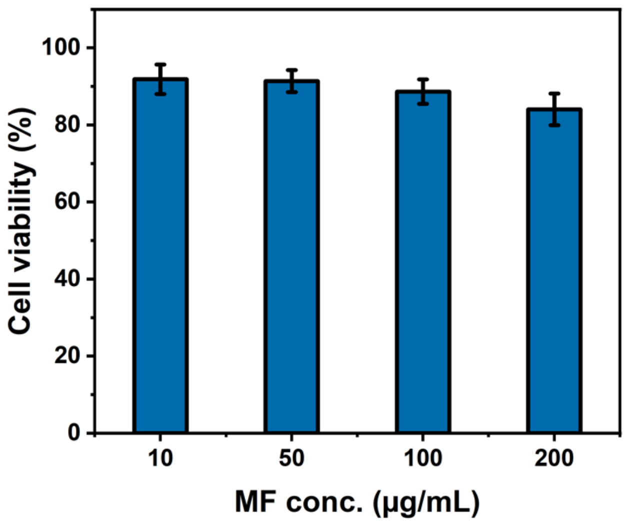

Figure S12. *In vitro* cytotoxicity of RM-GDL-MF on L929 cells.

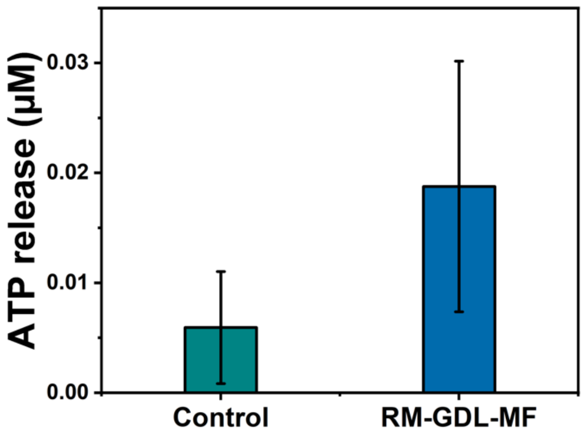

Figure S13. ATP release of RM-GDL-MF in MDA-MB-231 cells.

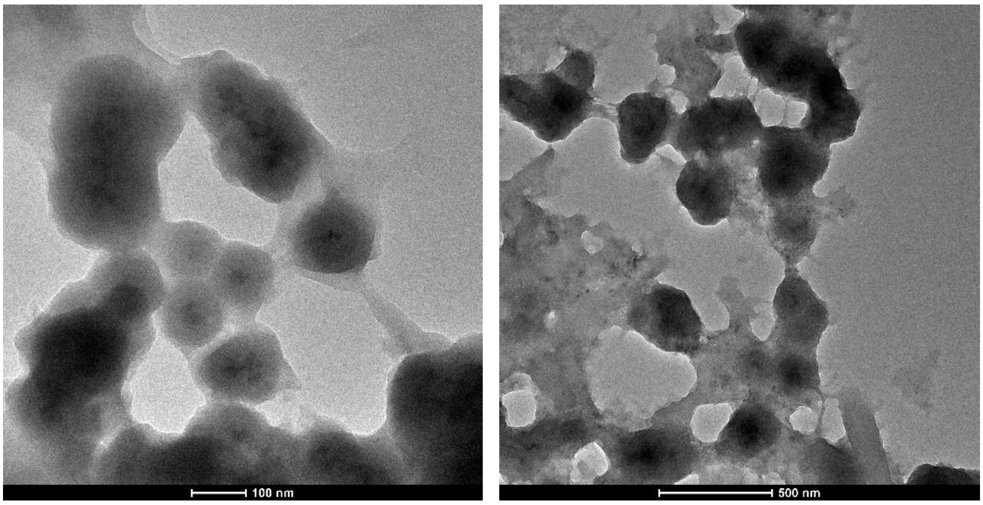

Figure S14. TEM images of R4-GDL-MF.

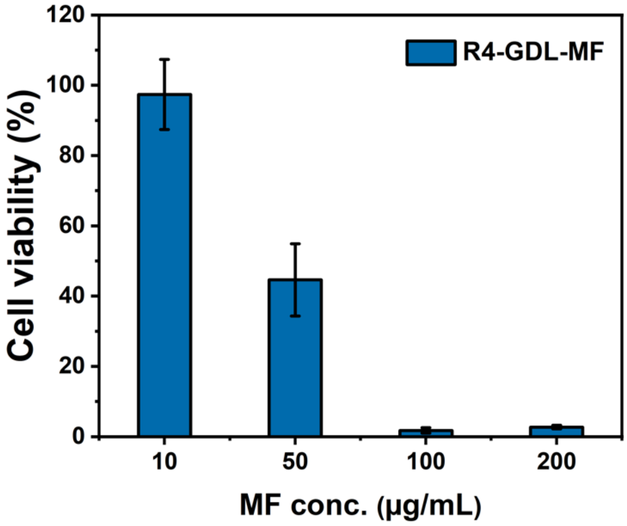

Figure S15. In vitro cytotoxicity of R4-GDL-MF on 4T1 cells.

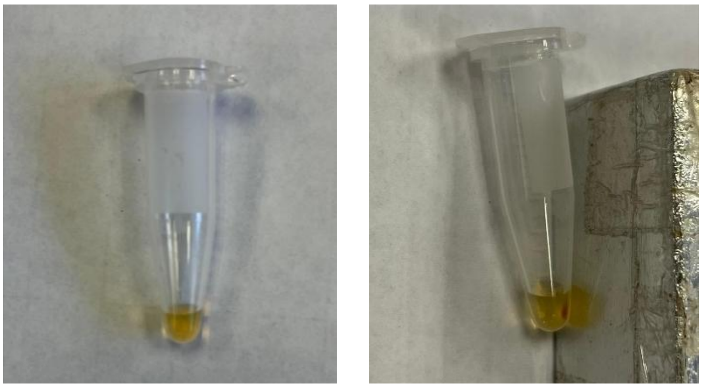

Figure S16. MF in EtOH before (left) and after (right) attaching to a magnet.

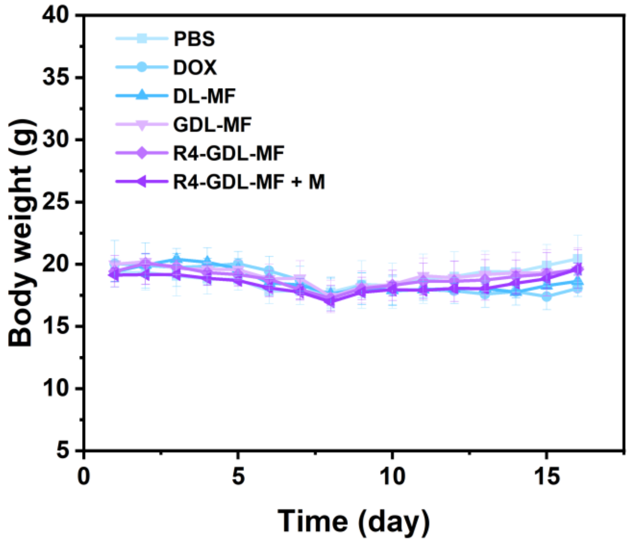

Figure S17. Body weight record of mice in different treatment groups.

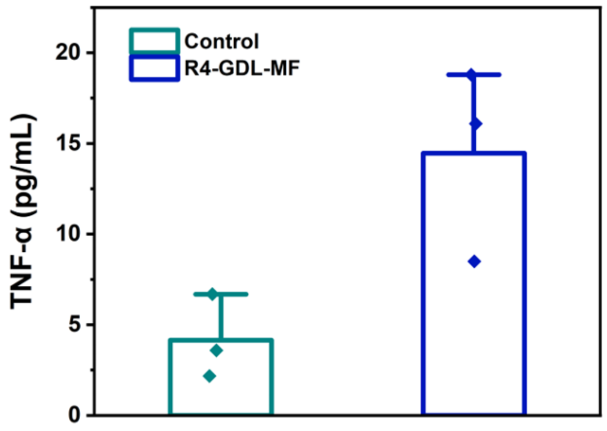

Figure S18. Serum TNF-α level of mice after 14-day of treatment with and without R4-GGDL-MF.

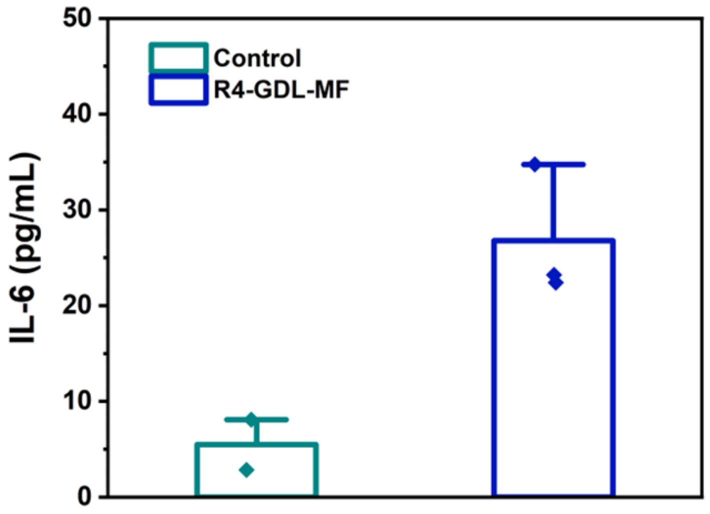

Figure S19. Serum IL-6 level in mice after 14-day treatment with and without R4-GGDL-MF.

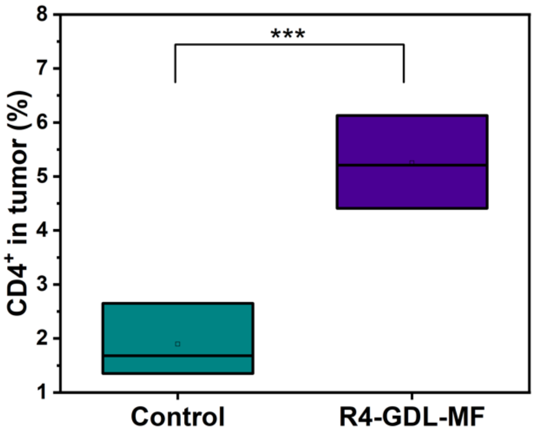

Figure S20. Quantification of intratumoral $CD4^{+}$ T cells by flow cytometry analysis.

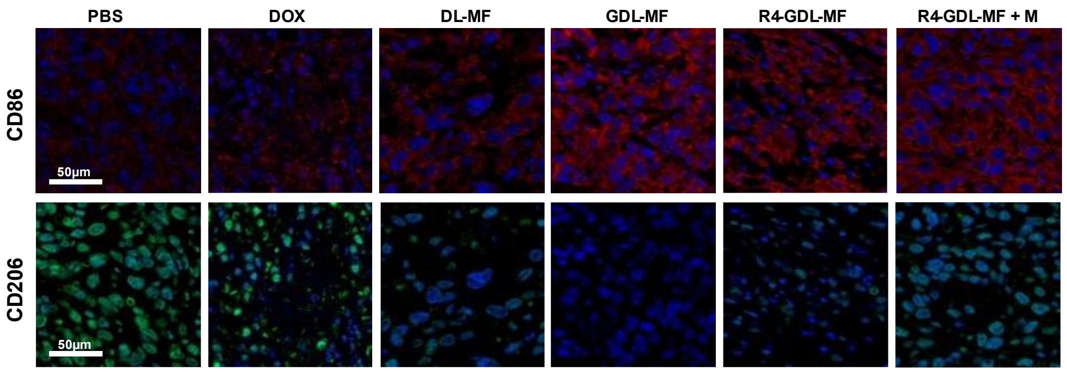

Figure S21. Immunofluorescence of CD86 and CD206 in tumor slice.

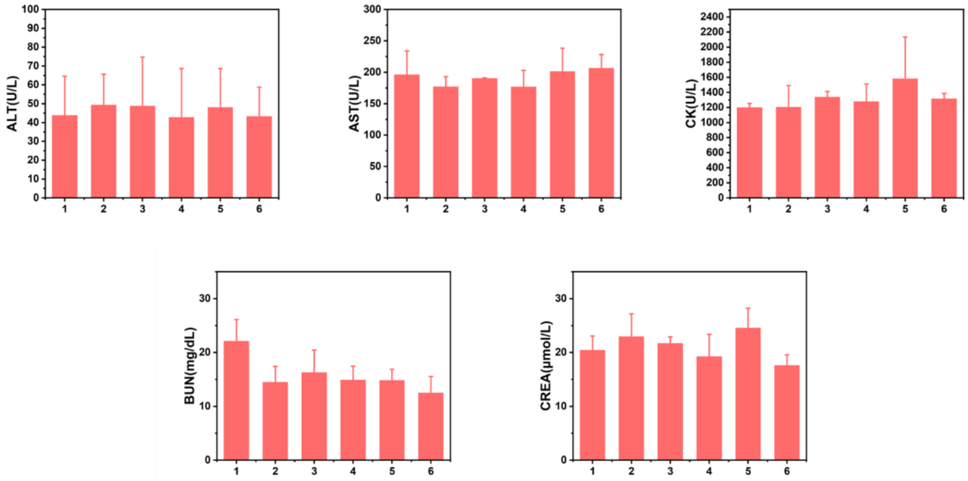

Figure S22. Blood biochemistry. Liver and kidney index upon treatment (1: PBS, 2: DOX, 3: DL-MF, 4: GDL-MF, 5: R4-GDL-MF, 6: R4-GDL-MF + M).

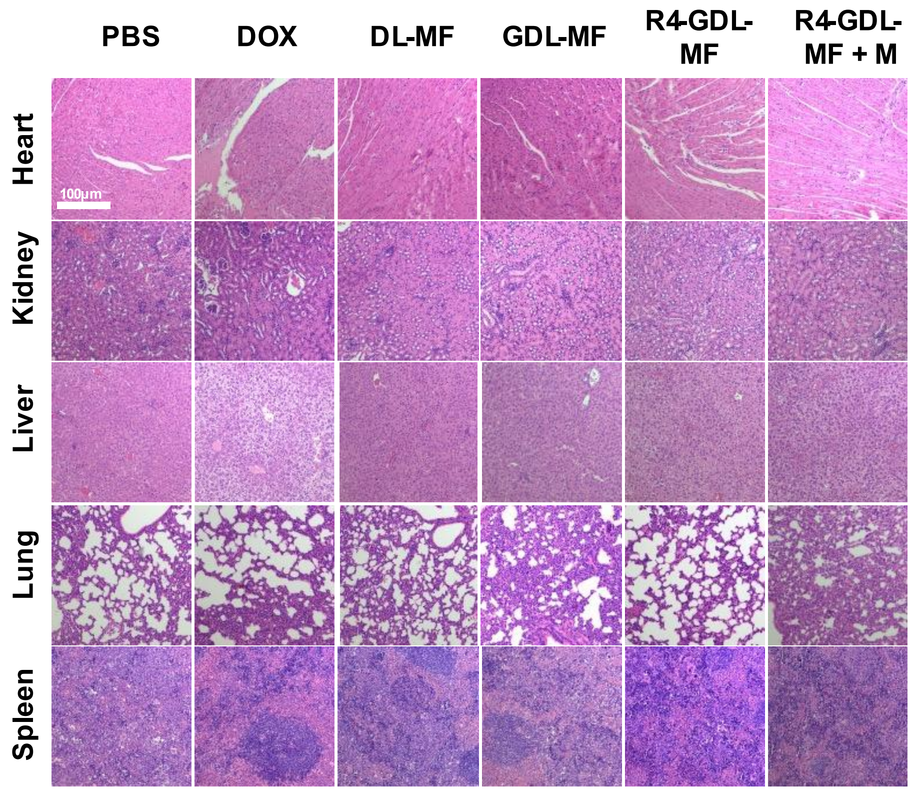

Figure S23. H&E staining of organs (heart, kidney, liver, lung and spleen) of different treated groups.
